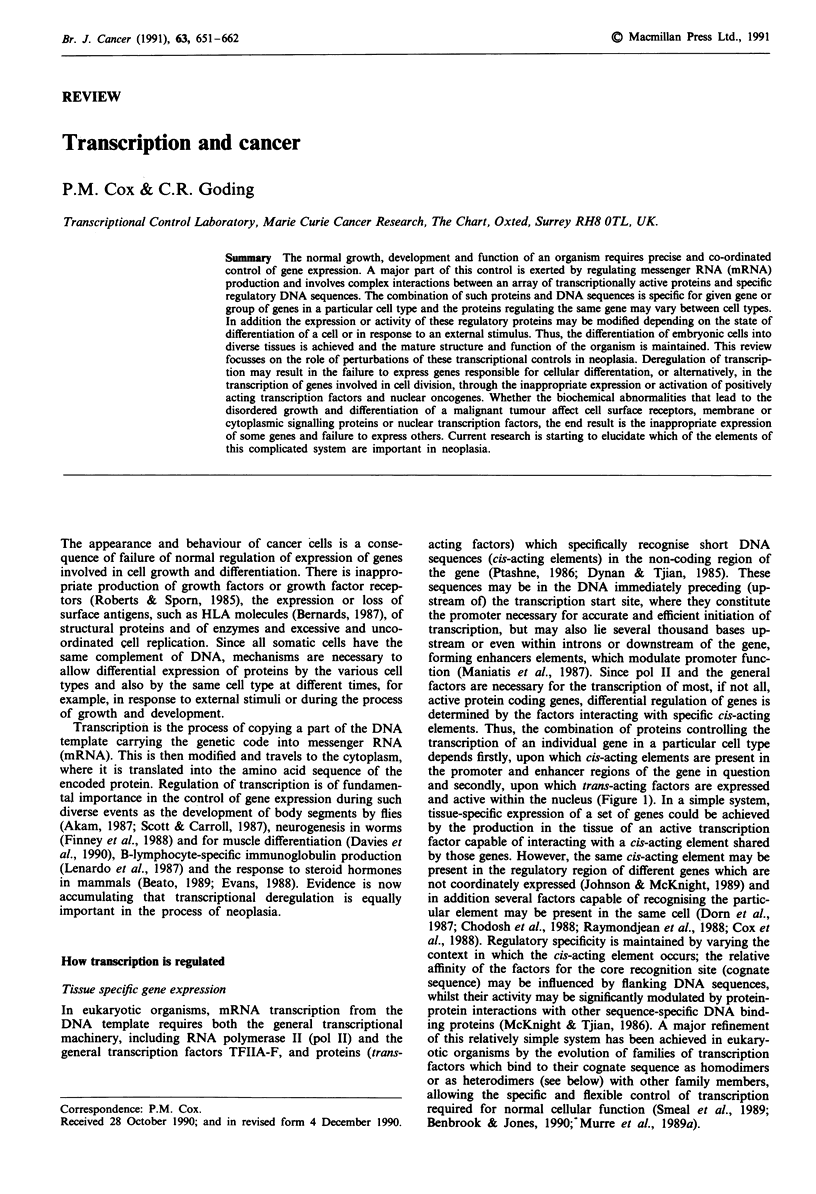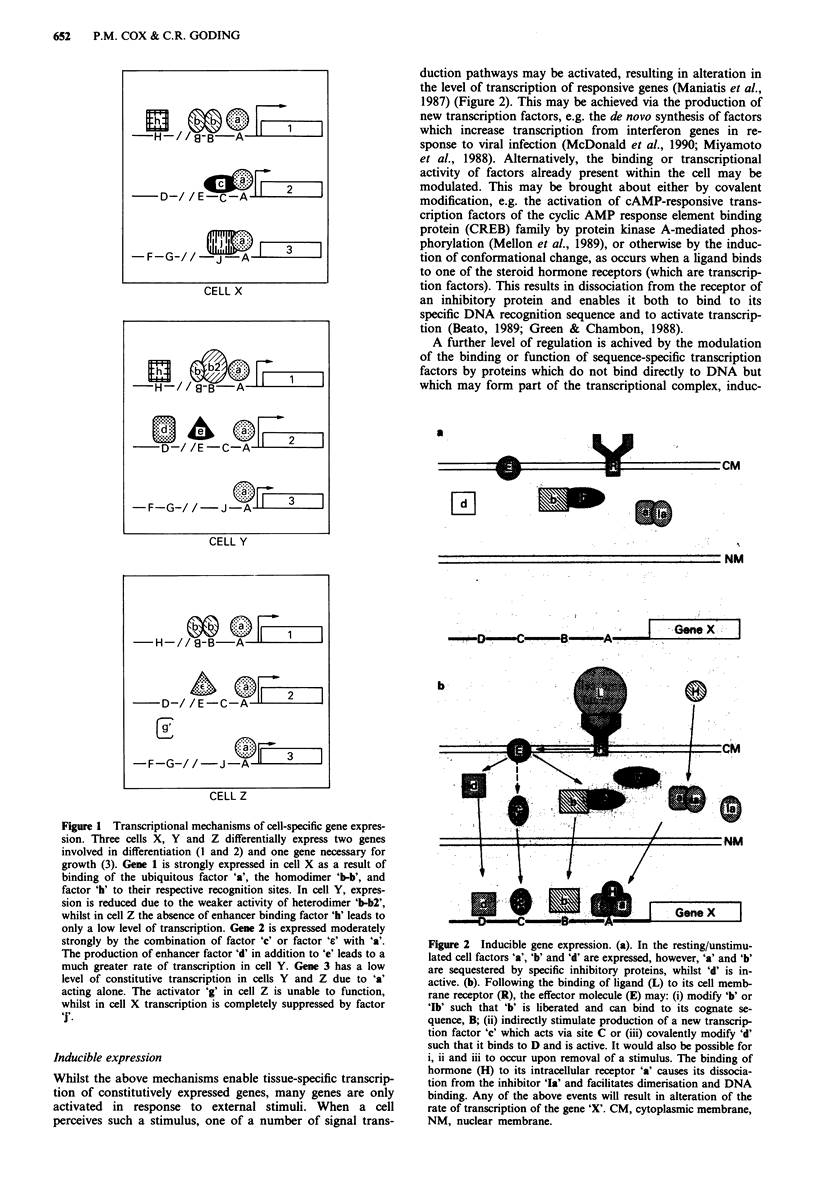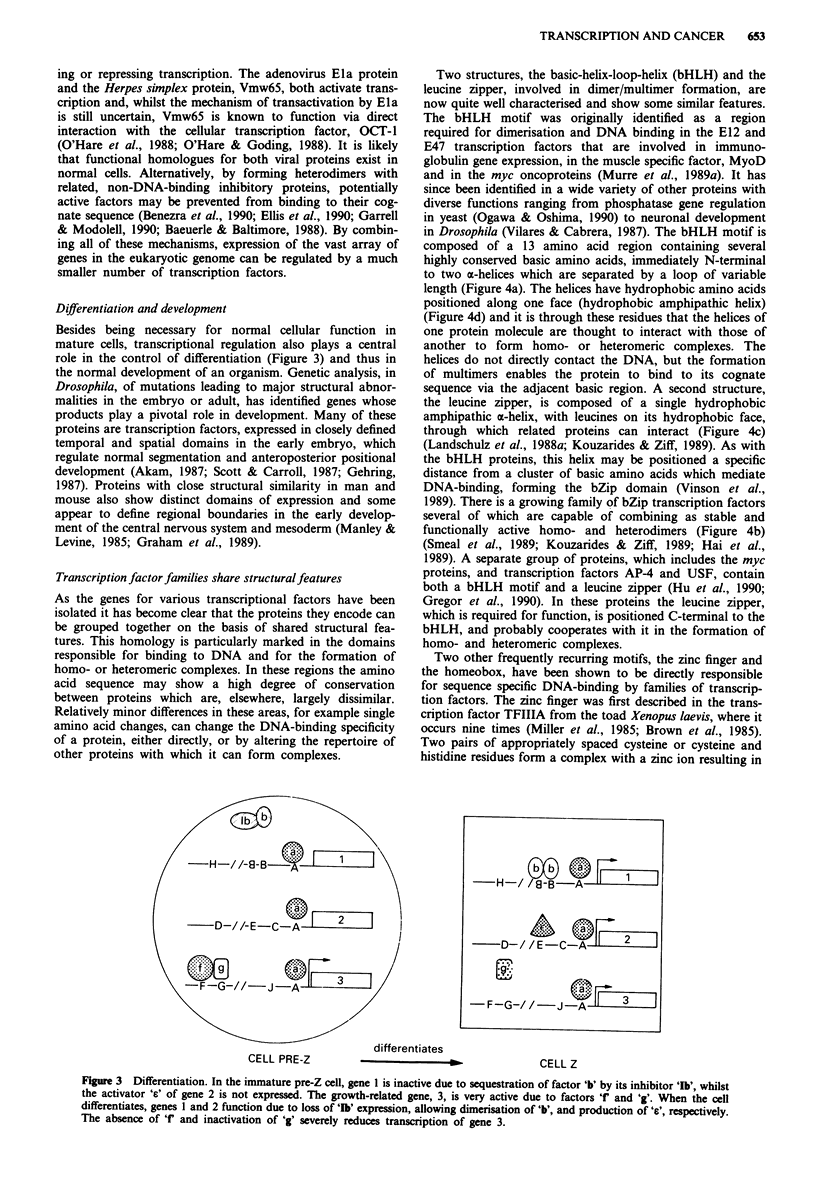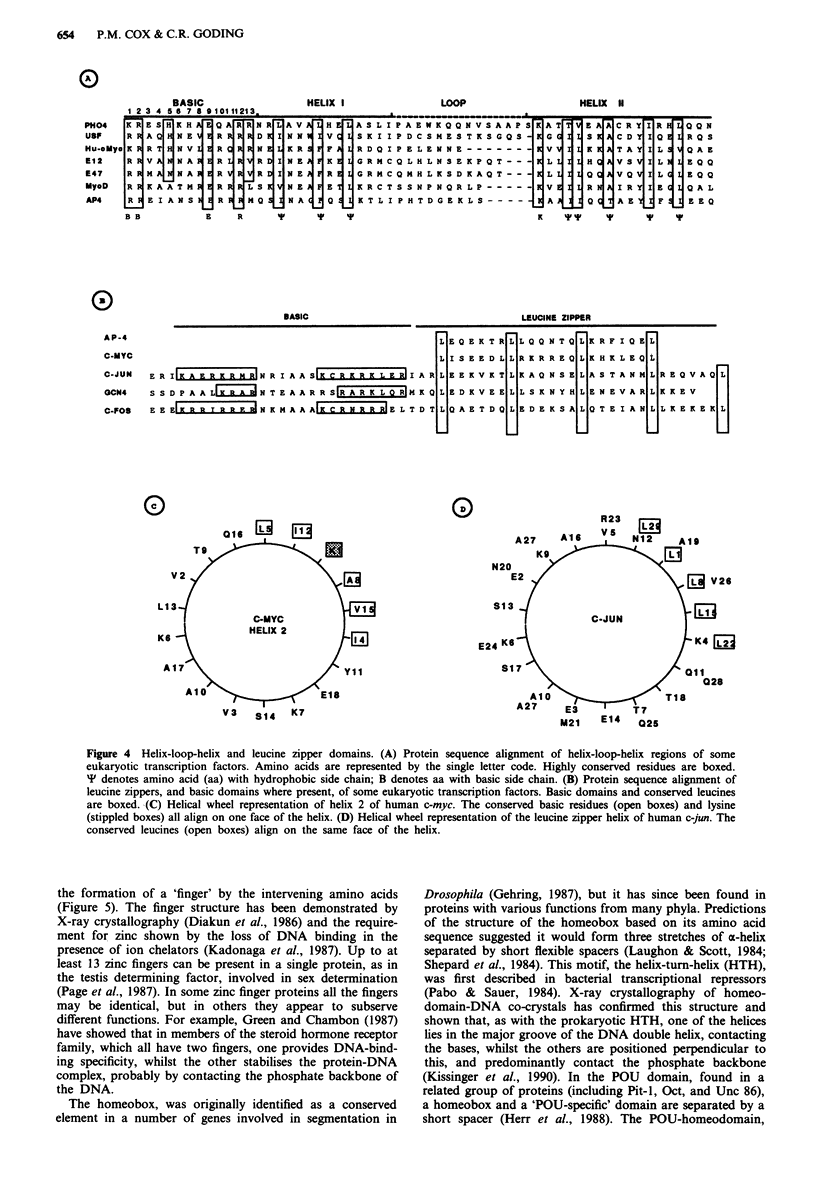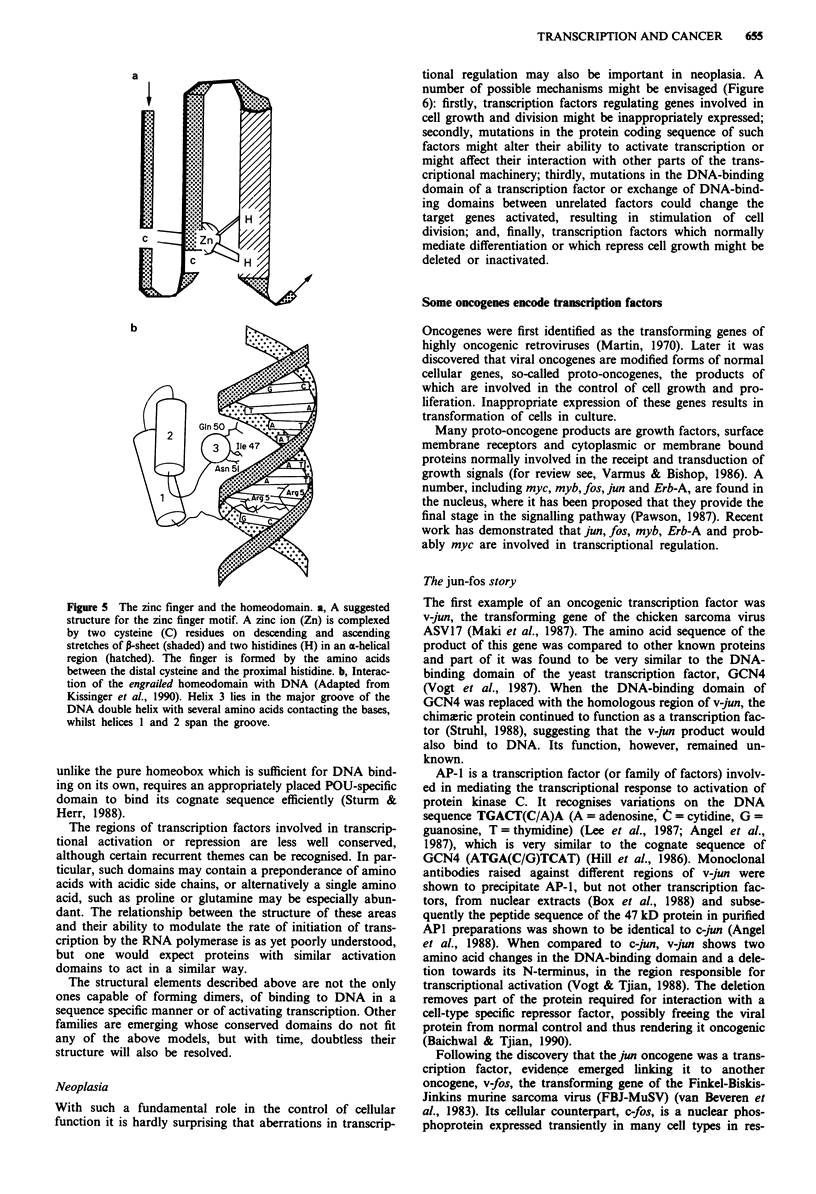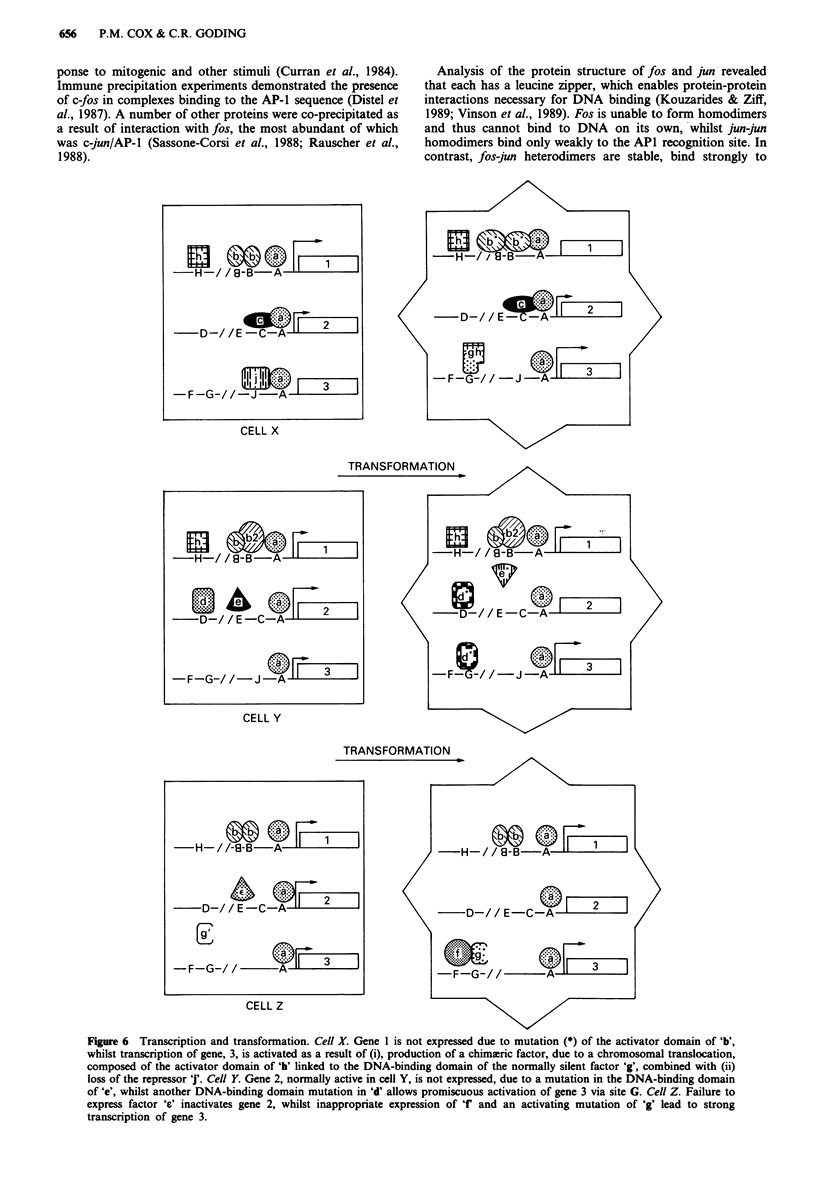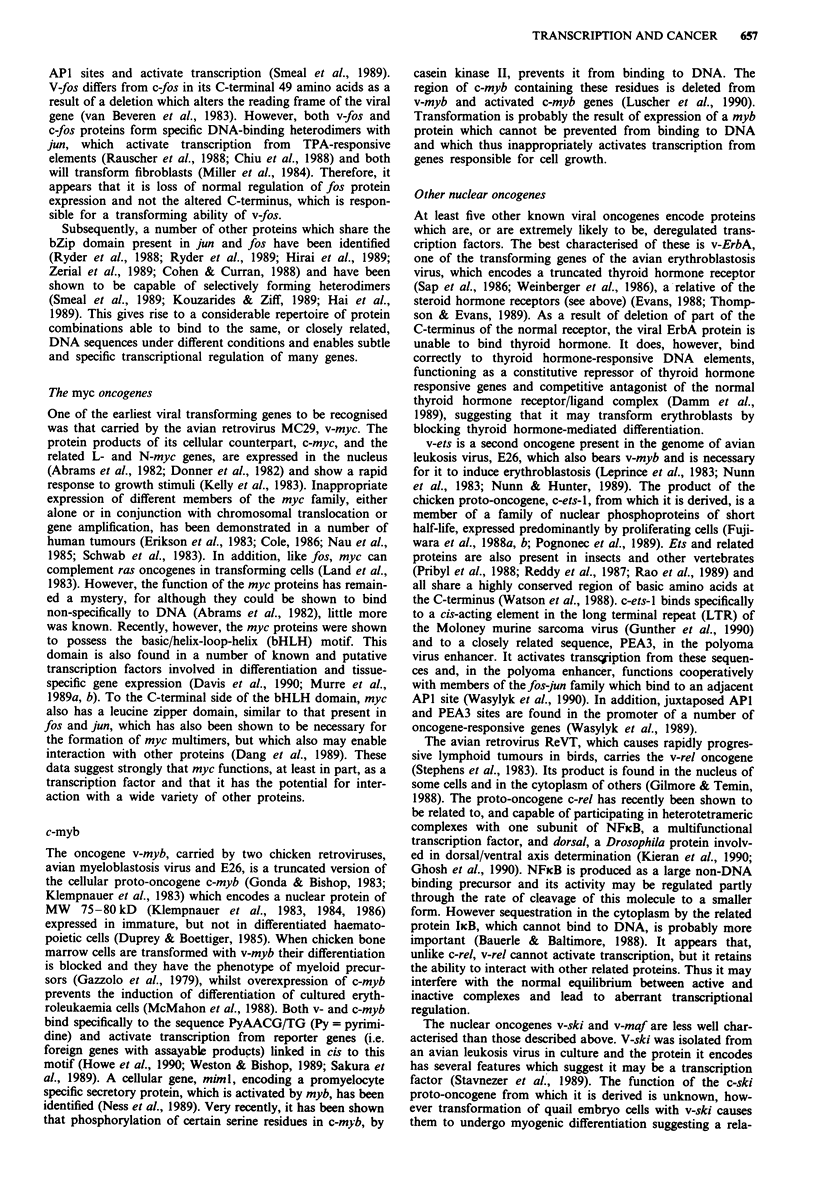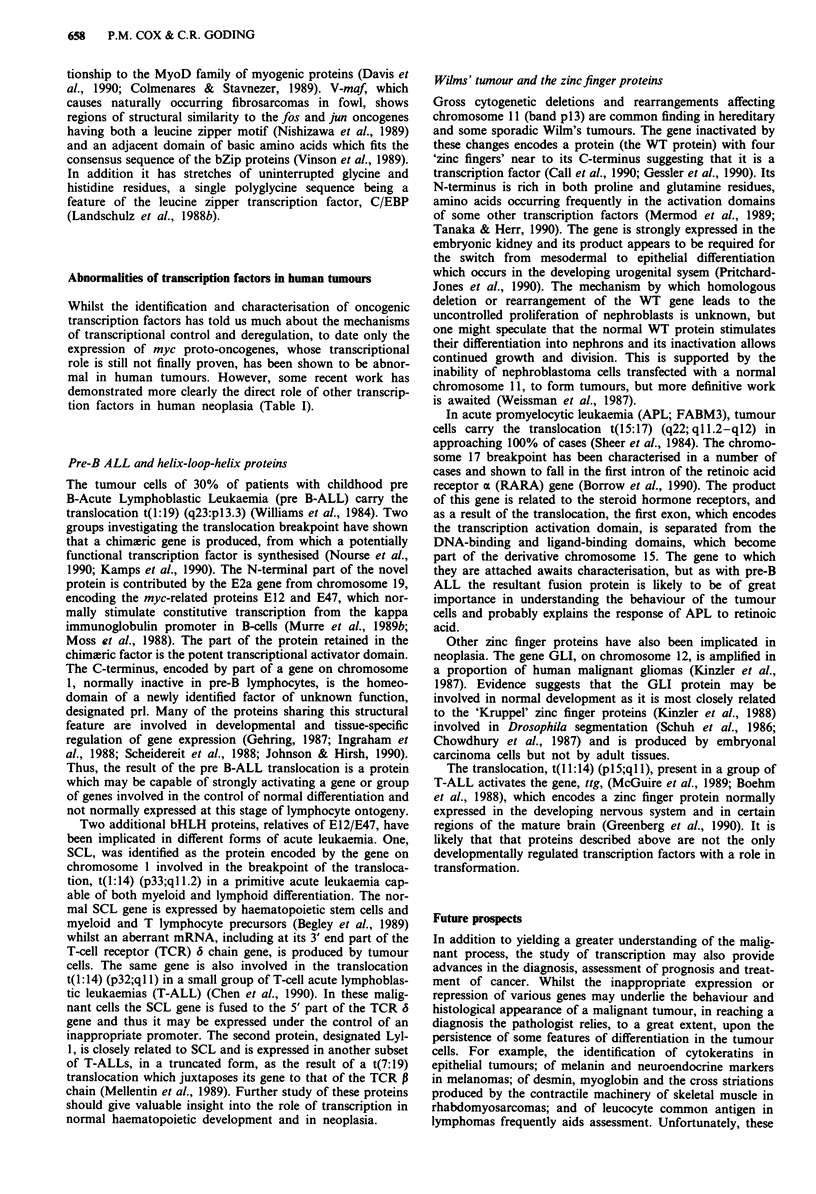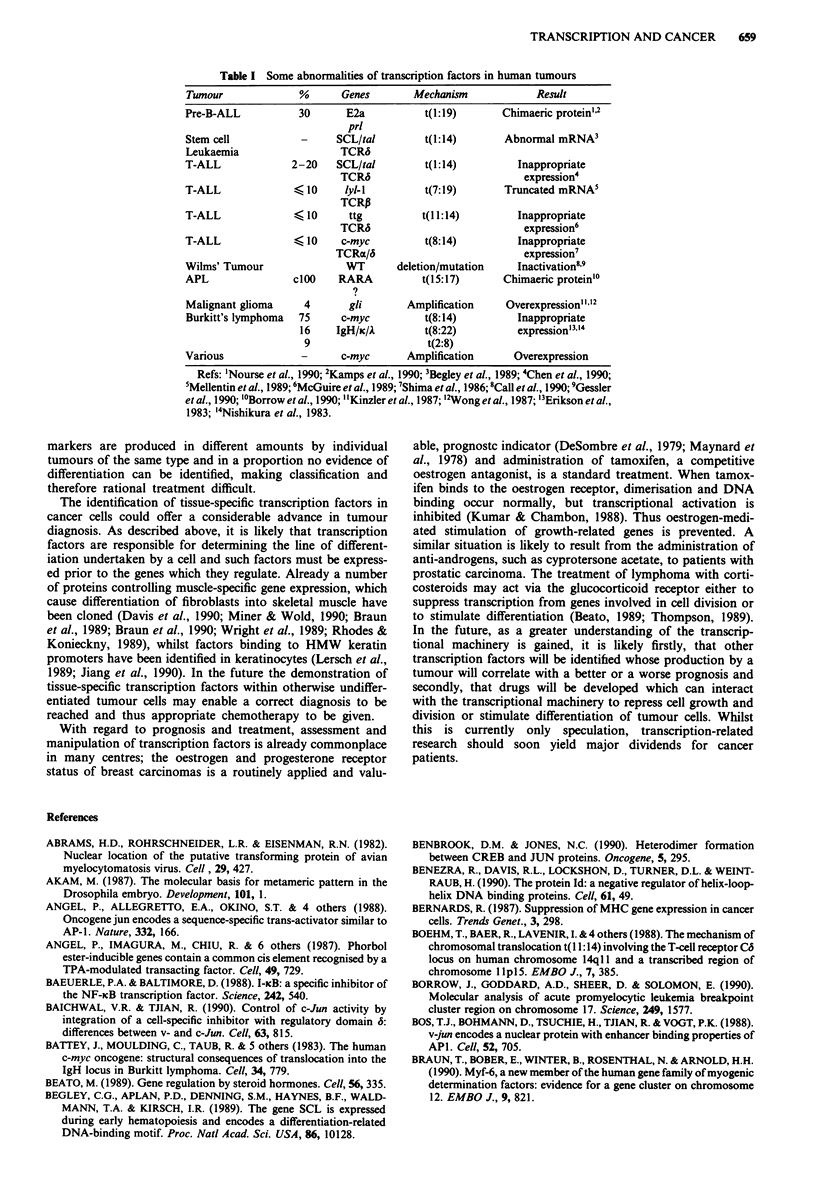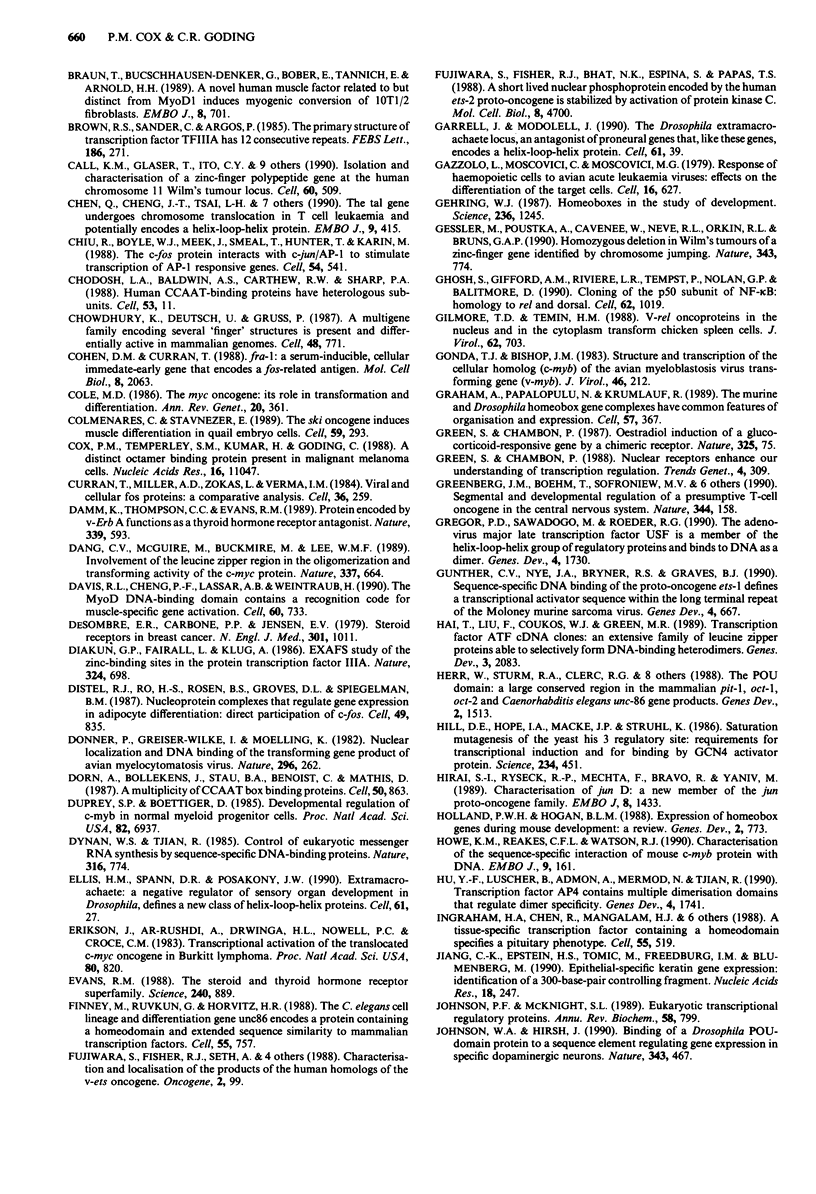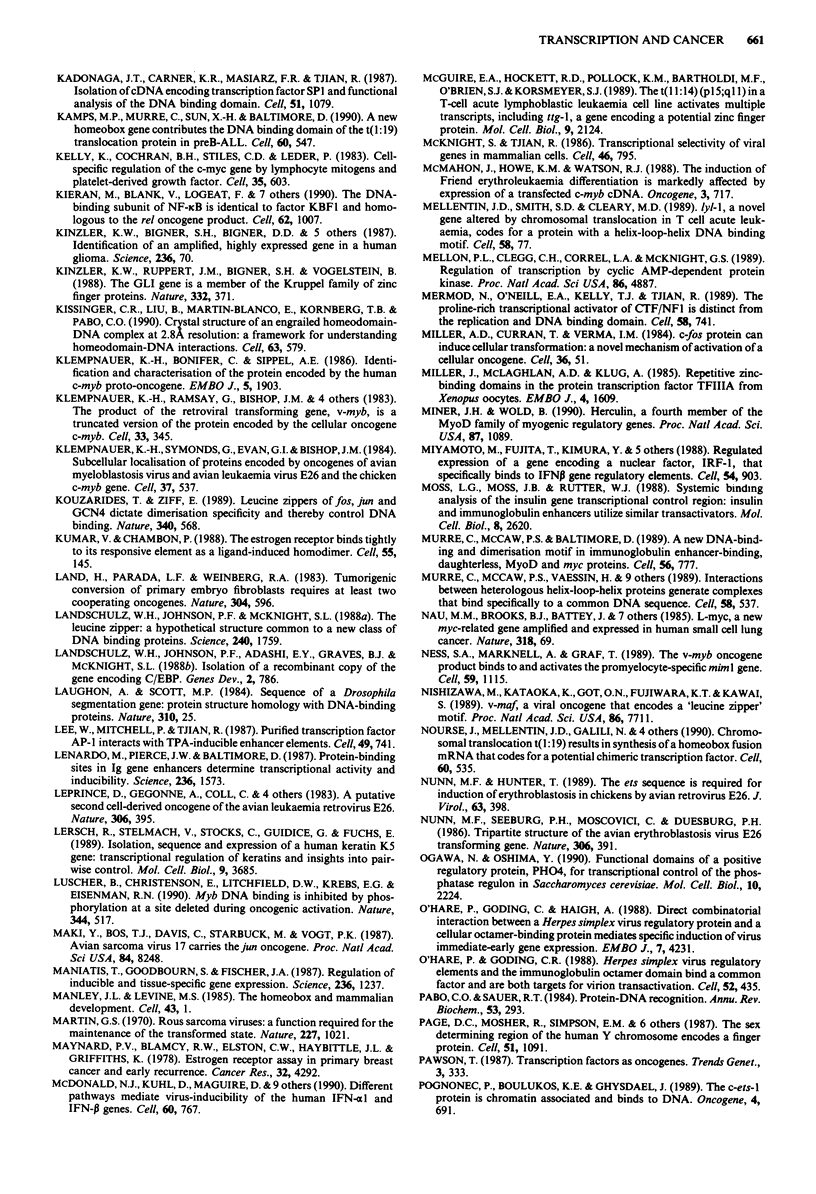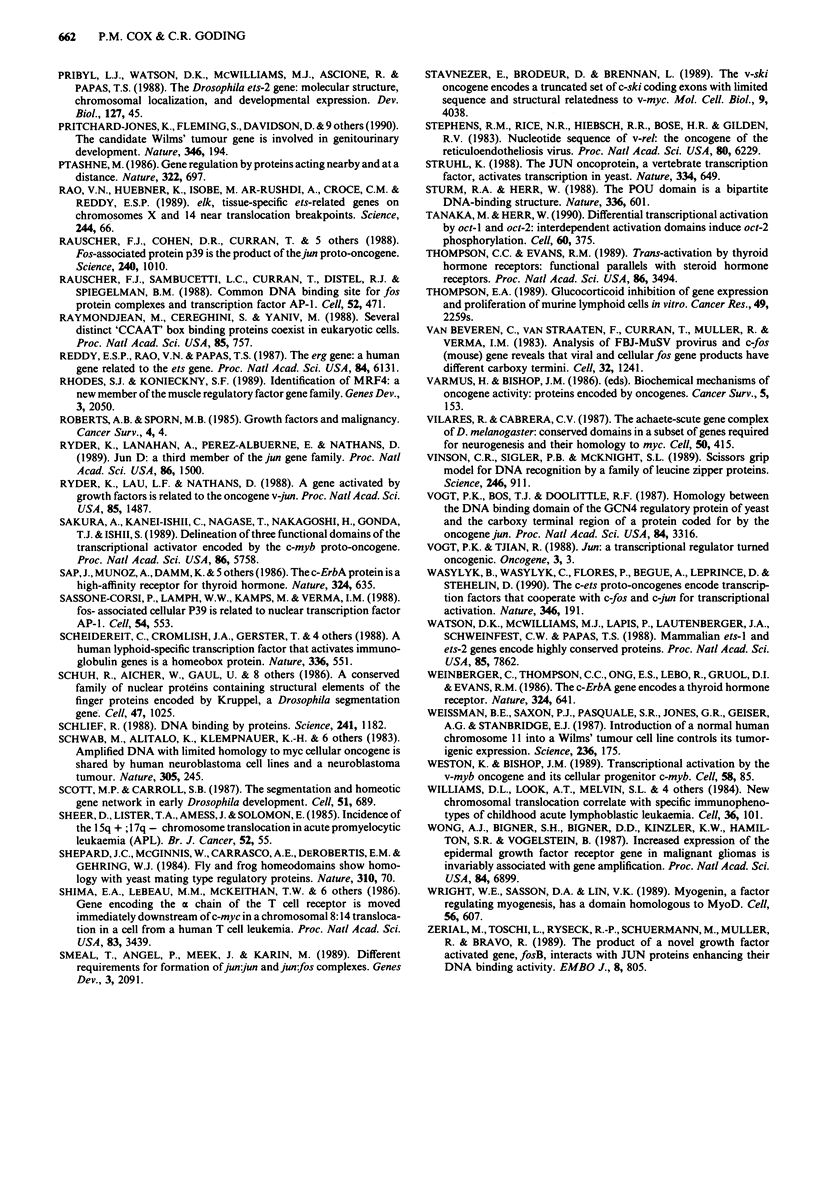# Transcription and cancer.

**DOI:** 10.1038/bjc.1991.151

**Published:** 1991-05

**Authors:** P. M. Cox, C. R. Goding

**Affiliations:** Transcriptional Control Laboratory, Marie Curie Cancer Research, Oxted, Surrey, UK.

## Abstract

The normal growth, development and function of an organism requires precise and co-ordinated control of gene expression. A major part of this control is exerted by regulating messenger RNA (mRNA) production and involves complex interactions between an array of transcriptionally active proteins and specific regulatory DNA sequences. The combination of such proteins and DNA sequences is specific for given gene or group of genes in a particular cell type and the proteins regulating the same gene may vary between cell types. In addition the expression or activity of these regulatory proteins may be modified depending on the state of differentiation of a cell or in response to an external stimulus. Thus, the differentiation of embryonic cells into diverse tissues is achieved and the mature structure and function of the organism is maintained. This review focusses on the role of perturbations of these transcriptional controls in neoplasia. Deregulation of transcription may result in the failure to express genes responsible for cellular differentiation, or alternatively, in the transcription of genes involved in cell division, through the inappropriate expression or activation of positively acting transcription factors and nuclear oncogenes. Whether the biochemical abnormalities that lead to the disordered growth and differentiation of a malignant tumour affect cell surface receptors, membrane or cytoplasmic signalling proteins or nuclear transcription factors, the end result is the inappropriate expression of some genes and failure to express others. Current research is starting to elucidate which of the elements of this complicated system are important in neoplasia.


*Br. J. Cancer* (1991), **63**, 651–662 © Macmillan Press Ltd., 1991

**REVIEW**

### Transcription and cancer

P.M. Cox & C.R. Goding

*Transcriptional Control Laboratory, Marie Curie Cancer Research, The Chart, Oxted, Surrey RH8 0TL, UK.*

**Summary** The normal growth, development and function of an organism requires precise and co-ordinated control of gene expression. A major part of this control is exerted by regulating messenger RNA (mRNA) production and involves complex interactions between an array of transcriptionally active proteins and specific regulatory DNA sequences. The combination of such proteins and DNA sequences is specific for given gene or group of genes in a particular cell type and the proteins regulating the same gene may vary between cell types. In addition the expression or activity of these regulatory proteins may be modified depending on the state of differentiation of a cell or in response to an external stimulus. Thus, the differentiation of embryonic cells into diverse tissues is achieved and the mature structure and function of the organism is maintained. This review focusses on the role of perturbations of these transcriptional controls in neoplasia. Deregulation of transcription may result in the failure to express genes responsible for cellular differentation, or alternatively, in the transcription of genes involved in cell division, through the inappropriate expression or activation of positively acting transcription factors and nuclear oncogenes. Whether the biochemical abnormalities that lead to the disordered growth and differentiation of a malignant tumour affect cell surface receptors, membrane or cytoplasmic signalling proteins or nuclear transcription factors, the end result is the inappropriate expression of some genes and failure to express others. Current research is starting to elucidate which of the elements of this complicated system are important in neoplasia.

The appearance and behaviour of cancer cells is a consequence of failure of normal regulation of expression of genes involved in cell growth and differentiation. There is inappropriate production of growth factors or growth factor receptors (Roberts & Sporn, 1985), the expression or loss of surface antigens, such as HLA molecules (Bernards, 1987), of structural proteins and of enzymes and excessive and uncoordinated cell replication. Since all somatic cells have the same complement of DNA, mechanisms are necessary to allow differential expression of proteins by the various cell types and also by the same cell type at different times, for example, in response to external stimuli or during the process of growth and development.

Transcription is the process of copying a part of the DNA template carrying the genetic code into messenger RNA (mRNA). This is then modified and travels to the cytoplasm, where it is translated into the amino acid sequence of the encoded protein. Regulation of transcription is of fundamental importance in the control of gene expression during such diverse events as the development of body segments by flies (Akam, 1987; Scott & Carroll, 1987), neurogenesis in worms (Finney *et al.*, 1988) and for muscle differentiation (Davies *et al.*, 1990), B-lymphocyte-specific immunoglobulin production (Lenardo *et al.*, 1987) and the response to steroid hormones in mammals (Beato, 1989; Evans, 1988). Evidence is now accumulating that transcriptional deregulation is equally important in the process of neoplasia.

### How transcription is regulated

### *Tissue specific gene expression*

In eukaryotic organisms, mRNA transcription from the DNA template requires both the general transcriptional machinery, including RNA polymerase II (pol II) and the general transcription factors TFIIA-F, and proteins (*trans*-acting factors) which specifically recognise short DNA sequences (*cis*-acting elements) in the non-coding region of the gene (Ptashne, 1986; Dynan & Tjian, 1985). These sequences may be in the DNA immediately preceding (upstream of) the transcription start site, where they constitute the promoter necessary for accurate and efficient initiation of transcription, but may also lie several thousand bases upstream or even within introns or downstream of the gene, forming enhancers elements, which modulate promoter function (Maniatis *et al.*, 1987). Since pol II and the general factors are necessary for the transcription of most, if not all, active protein coding genes, differential regulation of genes is determined by the factors interacting with specific *cis*-acting elements. Thus, the combination of proteins controlling the transcription of an individual gene in a particular cell type depends firstly, upon which *cis*-acting elements are present in the promoter and enhancer regions of the gene in question and secondly, upon which *trans*-acting factors are expressed and active within the nucleus (Figure 1). In a simple system, tissue-specific expression of a set of genes could be achieved by the production in the tissue of an active transcription factor capable of interacting with a *cis*-acting element shared by those genes. However, the same *cis*-acting element may be present in the regulatory region of different genes which are not coordinately expressed (Johnson & McKnight, 1989) and in addition several factors capable of recognising the particular element may be present in the same cell (Dorn *et al.*, 1987; Chodosh *et al.*, 1988; Raymondjean *et al.*, 1988; Cox *et al.*, 1988). Regulatory specificity is maintained by varying the context in which the *cis*-acting element occurs; the relative affinity of the factors for the core recognition site (cognate sequence) may be influenced by flanking DNA sequences, whilst their activity may be significantly modulated by protein-protein interactions with other sequence-specific DNA binding proteins (McKnight & Tjian, 1986). A major refinement of this relatively simple system has been achieved in eukaryotic organisms by the evolution of families of transcription factors which bind to their cognate sequence as homodimers or as heterodimers (see below) with other family members, allowing the specific and flexible control of transcription required for normal cellular function (Smeal *et al.*, 1989; Benbrook & Jones, 1990; Murre *et al.*, 1989*a*).

Correspondence: P.M. Cox.
Received 28 October 1990; and in revised form 4 December 1990.

**Figure 1** Transcriptional mechanisms of cell-specific gene expression. Three cells X, Y and Z differentially express two genes involved in differentiation (1 and 2) and one gene necessary for growth (3). **Gene 1** is strongly expressed in cell X as a result of binding of the ubiquitous factor '**a**', the homodimer '**b-b**', and factor '**h**' to their respective recognition sites. In cell Y, expression is reduced due to the weaker activity of heterodimer '**b-b2**', whilst in cell Z the absence of enhancer binding factor '**h**' leads to only a low level of transcription. **Gene 2** is expressed moderately strongly by the combination of factor '**c**' or factor 'ε' with '**a**'. The production of enhancer factor '**d**' in addition to '**e**' leads to a much greater rate of transcription in cell Y. **Gene 3** has a low level of constitutive transcription in cells Y and Z due to '**a**' acting alone. The activator '**g**' in cell Z is unable to function, whilst in cell X transcription is completely suppressed by factor '**j**'.

*Inducible expression*

Whilst the above mechanisms enable tissue-specific transcription of constitutively expressed genes, many genes are only activated in response to external stimuli. When a cell perceives such a stimulus, one of a number of signal transduction pathways may be activated, resulting in alteration in the level of transcription of responsive genes (Maniatis *et al.*, 1987) (Figure 2). This may be achieved via the production of new transcription factors, e.g. the *de novo* synthesis of factors which increase transcription from interferon genes in response to viral infection (McDonald *et al.*, 1990; Miyamoto *et al.*, 1988). Alternatively, the binding or transcriptional activity of factors already present within the cell may be modulated. This may be brought about either by covalent modification, e.g. the activation of cAMP-responsive transcription factors of the cyclic AMP response element binding protein (CREB) family by protein kinase A-mediated phosphorylation (Mellon *et al.*, 1989), or otherwise by the induction of conformational change, as occurs when a ligand binds to one of the steroid hormone receptors (which are transcription factors). This results in dissociation from the receptor of an inhibitory protein and enables it both to bind to its specific DNA recognition sequence and to activate transcription (Beato, 1989; Green & Chambon, 1988).

A further level of regulation is achived by the modulation of the binding or function of sequence-specific transcription factors by proteins which do not bind directly to DNA but which may form part of the transcriptional complex, induc-

**Figure 2** Inducible gene expression. (**a**). In the resting/unstimulated cell factors '**a**', '**b**' and '**d**' are expressed, however, '**a**' and '**b**' are sequestered by specific inhibitory proteins, whilst '**d**' is inactive. (**b**). Following the binding of ligand (**L**) to its cell membrane receptor (**R**), the effector molecule (**E**) may: (i) modify '**b**' or '**Ib**' such that '**b**' is liberated and can bind to its cognate sequence, **B**; (ii) indirectly stimulate production of a new transcription factor '**c**' which acts via site **C** or (iii) covalently modify '**d**' such that it binds to **D** and is active. It would also be possible for i, ii and iii to occur upon removal of a stimulus. The binding of hormone (**H**) to its intracellular receptor '**a**' causes its dissociation from the inhibitor '**Ia**' and facilitates dimerisation and DNA binding. Any of the above events will result in alteration of the rate of transcription of the gene '**X**'. CM, cytoplasmic membrane, NM, nuclear membrane.

ing or repressing transcription. The adenovirus E1a protein and the *Herpes simplex* protein, Vmw65, both activate transcription and, whilst the mechanism of transactivation by E1a is still uncertain, Vmw65 is known to function via direct interaction with the cellular transcription factor, OCT-1 (O'Hare *et al.*, 1988; O'Hare & Goding, 1988). It is likely that functional homologues for both viral proteins exist in normal cells. Alternatively, by forming heterodimers with related, non-DNA-binding inhibitory proteins, potentially active factors may be prevented from binding to their cognate sequence (Benezra *et al.*, 1990; Ellis *et al.*, 1990; Garrell & Modolell, 1990; Baeuerle & Baltimore, 1988). By combining all of these mechanisms, expression of the vast array of genes in the eukaryotic genome can be regulated by a much smaller number of transcription factors.

### *Differentiation and development*

Besides being necessary for normal cellular function in mature cells, transcriptional regulation also plays a central role in the control of differentiation (Figure 3) and thus in the normal development of an organism. Genetic analysis, in *Drosophila*, of mutations leading to major structural abnormalities in the embryo or adult, has identified genes whose products play a pivotal role in development. Many of these proteins are transcription factors, expressed in closely defined temporal and spatial domains in the early embryo, which regulate normal segmentation and anteroposterior positional development (Akam, 1987; Scott & Carroll, 1987; Gehring, 1987). Proteins with close structural similarity in man and mouse also show distinct domains of expression and some appear to define regional boundaries in the early development of the central nervous system and mesoderm (Manley & Levine, 1985; Graham *et al.*, 1989).

### *Transcription factor families share structural features*

As the genes for various transcriptional factors have been isolated it has become clear that the proteins they encode can be grouped together on the basis of shared structural features. This homology is particularly marked in the domains responsible for binding to DNA and for the formation of homo- or heteromeric complexes. In these regions the amino acid sequence may show a high degree of conservation between proteins which are, elsewhere, largely dissimilar. Relatively minor differences in these areas, for example single amino acid changes, can change the DNA-binding specificity of a protein, either directly, or by altering the repertoire of other proteins with which it can form complexes.

Two structures, the basic-helix-loop-helix (bHLH) and the leucine zipper, involved in dimer/multimer formation, are now quite well characterised and show some similar features. The bHLH motif was originally identified as a region required for dimerisation and DNA binding in the E12 and E47 transcription factors that are involved in immunoglobulin gene expression, in the muscle specific factor, MyoD and in the *myc* oncoproteins (Murre *et al.*, 1989*a*). It has since been identified in a wide variety of other proteins with diverse functions ranging from phosphatase gene regulation in yeast (Ogawa & Oshima, 1990) to neuronal development in *Drosophila* (Vilares & Cabrera, 1987). The bHLH motif is composed of a 13 amino acid region containing several highly conserved basic amino acids, immediately N-terminal to two α-helices which are separated by a loop of variable length (Figure 4a). The helices have hydrophobic amino acids positioned along one face (hydrophobic amphipathic helix) (Figure 4d) and it is through these residues that the helices of one protein molecule are thought to interact with those of another to form homo- or heteromeric complexes. The helices do not directly contact the DNA, but the formation of multimers enables the protein to bind to its cognate sequence via the adjacent basic region. A second structure, the leucine zipper, is composed of a single hydrophobic amphipathic α-helix, with leucines on its hydrophobic face, through which related proteins can interact (Figure 4c) (Landschulz *et al.*, 1988*a*; Kouzarides & Ziff, 1989). As with the bHLH proteins, this helix may be positioned a specific distance from a cluster of basic amino acids which mediate DNA-binding, forming the bZip domain (Vinson *et al.*, 1989). There is a growing family of bZip transcription factors several of which are capable of combining as stable and functionally active homo- and heterodimers (Figure 4b) (Smeal *et al.*, 1989; Kouzarides & Ziff, 1989; Hai *et al.*, 1989). A separate group of proteins, which includes the *myc* proteins, and transcription factors AP-4 and USF, contain both a bHLH motif and a leucine zipper (Hu *et al.*, 1990; Gregor *et al.*, 1990). In these proteins the leucine zipper, which is required for function, is positioned C-terminal to the bHLH, and probably cooperates with it in the formation of homo- and heteromeric complexes.

Two other frequently recurring motifs, the zinc finger and the homeobox, have been shown to be directly responsible for sequence specific DNA-binding by families of transcription factors. The zinc finger was first described in the transcription factor TFIIIA from the toad *Xenopus laevis*, where it occurs nine times (Miller *et al.*, 1985; Brown *et al.*, 1985). Two pairs of appropriately spaced cysteine or cysteine and histidine residues form a complex with a zinc ion resulting in

**Figure 3** Differentiation. In the immature pre-Z cell, gene 1 is inactive due to sequestration of factor '**b**' by its inhibitor '**Ib**', whilst the activator 'ε' of gene 2 is not expressed. The growth-related gene, 3, is very active due to factors '**f**' and '**g**'. When the cell differentiates, genes 1 and 2 function due to loss of '**Ib**' expression, allowing dimerisation of '**b**', and production of 'ε', respectively. The absence of '**f**' and inactivation of '**g**' severely reduces transcription of gene 3.

**Figure 4** Helix-loop-helix and leucine zipper domains. (A) Protein sequence alignment of helix-loop-helix regions of some eukaryotic transcription factors. Amino acids are represented by the single letter code. Highly conserved residues are boxed. Ψ denotes amino acid (aa) with hydrophobic side chain; B denotes aa with basic side chain. (B) Protein sequence alignment of leucine zippers, and basic domains where present, of some eukaryotic transcription factors. Basic domains and conserved leucines are boxed. (C) Helical wheel representation of helix 2 of human c-*myc*. The conserved basic residues (open boxes) and lysine (stippled boxes) all align on one face of the helix. (D) Helical wheel representation of the leucine zipper helix of human c-*jun*. The conserved leucines (open boxes) align on the same face of the helix.

the formation of a 'finger' by the intervening amino acids (Figure 5). The finger structure has been demonstrated by X-ray crystallography (Diakun *et al*., 1986) and the requirement for zinc shown by the loss of DNA binding in the presence of ion chelators (Kadonaga *et al*., 1987). Up to at least 13 zinc fingers can be present in a single protein, as in the testis determining factor, involved in sex determination (Page *et al*., 1987). In some zinc finger proteins all the fingers may be identical, but in others they appear to subserve different functions. For example, Green and Chambon (1987) have showed that in members of the steroid hormone receptor family, which all have two fingers, one provides DNA-binding specificity, whilst the other stabilises the protein-DNA complex, probably by contacting the phosphate backbone of the DNA.

The homeobox, was originally identified as a conserved element in a number of genes involved in segmentation in *Drosophila* (Gehring, 1987), but it has since been found in proteins with various functions from many phyla. Predictions of the structure of the homeobox based on its amino acid sequence suggested it would form three stretches of α-helix separated by short flexible spacers (Laughon & Scott, 1984; Shepard *et al*., 1984). This motif, the helix-turn-helix (HTH), was first described in bacterial transcriptional repressors (Pabo & Sauer, 1984). X-ray crystallography of homeodomain-DNA co-crystals has confirmed this structure and shown that, as with the prokaryotic HTH, one of the helices lies in the major groove of the DNA double helix, contacting the bases, whilst the others are positioned perpendicular to this, and predominantly contact the phosphate backbone (Kissinger *et al*., 1990). In the POU domain, found in a related group of proteins (including Pit-1, Oct, and Unc 86), a homeobox and a 'POU-specific' domain are separated by a short spacer (Herr *et al*., 1988). The POU-homeodomain,

**Figure 5** The zinc finger and the homeodomain. **a**, A suggested structure for the zinc finger motif. A zinc ion (Zn) is complexed by two cysteine (C) residues on descending and ascending stretches of β-sheet (shaded) and two histidines (H) in an α-helical region (hatched). The finger is formed by the amino acids between the distal cysteine and the proximal histidine. **b**, Interaction of the *engrailed* homeodomain with DNA (Adapted from Kissinger *et al*., 1990). Helix 3 lies in the major groove of the DNA double helix with several amino acids contacting the bases, whilst helices 1 and 2 span the groove.

unlike the pure homeobox which is sufficient for DNA binding on its own, requires an appropriately placed POU-specific domain to bind its cognate sequence efficiently (Sturm & Herr, 1988).

The regions of transcription factors involved in transcriptional activation or repression are less well conserved, although certain recurrent themes can be recognised. In particular, such domains may contain a preponderance of amino acids with acidic side chains, or alternatively a single amino acid, such as proline or glutamine may be especially abundant. The relationship between the structure of these areas and their ability to modulate the rate of initiation of transcription by the RNA polymerase is as yet poorly understood, but one would expect proteins with similar activation domains to act in a similar way.

The structural elements described above are not the only ones capable of forming dimers, of binding to DNA in a sequence specific manner or of activating transcription. Other families are emerging whose conserved domains do not fit any of the above models, but with time, doubtless their structure will also be resolved.

### *Neoplasia*

With such a fundamental role in the control of cellular function it is hardly surprising that aberrations in transcriptional regulation may also be important in neoplasia. A number of possible mechanisms might be envisaged (Figure 6): firstly, transcription factors regulating genes involved in cell growth and division might be inappropriately expressed; secondly, mutations in the protein coding sequence of such factors might alter their ability to activate transcription or might affect their interaction with other parts of the transcriptional machinery; thirdly, mutations in the DNA-binding domain of a transcription factor or exchange of DNA-binding domains between unrelated factors could change the target genes activated, resulting in stimulation of cell division; and, finally, transcription factors which normally mediate differentiation or which repress cell growth might be deleted or inactivated.

### Some oncogenes encode transcription factors

Oncogenes were first identified as the transforming genes of highly oncogenic retroviruses (Martin, 1970). Later it was discovered that viral oncogenes are modified forms of normal cellular genes, so-called proto-oncogenes, the products of which are involved in the control of cell growth and proliferation. Inappropriate expression of these genes results in transformation of cells in culture.

Many proto-oncogene products are growth factors, surface membrane receptors and cytoplasmic or membrane bound proteins normally involved in the receipt and transduction of growth signals (for review see, Varmus & Bishop, 1986). A number, including *myc*, *myb*, *fos*, *jun* and *Erb*-A, are found in the nucleus, where it has been proposed that they provide the final stage in the signalling pathway (Pawson, 1987). Recent work has demonstrated that *jun*, *fos*, *myb*, *Erb*-A and probably *myc* are involved in transcriptional regulation.

### *The* jun-fos *story*

The first example of an oncogenic transcription factor was v-*jun*, the transforming gene of the chicken sarcoma virus ASV17 (Maki *et al*., 1987). The amino acid sequence of the product of this gene was compared to other known proteins and part of it was found to be very similar to the DNA-binding domain of the yeast transcription factor, GCN4 (Vogt *et al*., 1987). When the DNA-binding domain of GCN4 was replaced with the homologous region of v-*jun*, the chimæric protein continued to function as a transcription factor (Struhl, 1988), suggesting that the v-*jun* product would also bind to DNA. Its function, however, remained unknown.

AP-1 is a transcription factor (or family of factors) involved in mediating the transcriptional response to activation of protein kinase C. It recognises variations on the DNA sequence **TGACT(C/A)A** (A = adenosine, C = cytidine, G = guanosine, T = thymidine) (Lee *et al*., 1987; Angel *et al*., 1987), which is very similar to the cognate sequence of GCN4 (ATGA(C/G)TCAT) (Hill *et al*., 1986). Monoclonal antibodies raised against different regions of v-*jun* were shown to precipitate AP-1, but not other transcription factors, from nuclear extracts (Box *et al*., 1988) and subsequently the peptide sequence of the 47 kD protein in purified AP1 preparations was shown to be identical to c-*jun* (Angel *et al*., 1988). When compared to c-*jun*, v-*jun* shows two amino acid changes in the DNA-binding domain and a deletion towards its N-terminus, in the region responsible for transcriptional activation (Vogt & Tjian, 1988). The deletion removes part of the protein required for interaction with a cell-type specific repressor factor, possibly freeing the viral protein from normal control and thus rendering it oncogenic (Baichwal & Tjian, 1990).

Following the discovery that the *jun* oncogene was a transcription factor, evidence emerged linking it to another oncogene, v-*fos*, the transforming gene of the Finkel-Biskis-Jinkins murine sarcoma virus (FBJ-MuSV) (van Beveren *et al*., 1983). Its cellular counterpart, c-*fos*, is a nuclear phosphoprotein expressed transiently in many cell types in response to mitogenic and other stimuli (Curran *et al.*, 1984). Immune precipitation experiments demonstrated the presence of c-*fos* in complexes binding to the AP-1 sequence (Distel *et al.*, 1987). A number of other proteins were co-precipitated as a result of interaction with *fos*, the most abundant of which was c-*jun*/AP-1 (Sassone-Corsi *et al.*, 1988; Rauscher *et al.*, 1988).

Analysis of the protein structure of *fos* and *jun* revealed that each has a leucine zipper, which enables protein-protein interactions necessary for DNA binding (Kouzarides & Ziff, 1989; Vinson *et al.*, 1989). *Fos* is unable to form homodimers and thus cannot bind to DNA on its own, whilst *jun-jun* homodimers bind only weakly to the AP1 recognition site. In contrast, *fos-jun* heterodimers are stable, bind strongly to

**Figure 6** Transcription and transformation. *Cell X.* Gene 1 is not expressed due to mutation (*) of the activator domain of '**b**', whilst transcription of gene, 3, is activated as a result of (i), production of a chimæric factor, due to a chromosomal translocation, composed of the activator domain of '**h**' linked to the DNA-binding domain of the normally silent factor '**g**', combined with (ii) loss of the repressor '**j**'. *Cell Y.* Gene 2, normally active in cell Y, is not expressed, due to a mutation in the DNA-binding domain of '**e**', whilst another DNA-binding domain mutation in '**d**' allows promiscuous activation of gene 3 via site **G**. *Cell Z.* Failure to express factor 'ε' inactivates gene 2, whilst inappropriate expression of '**f**' and an activating mutation of '**g**' lead to strong transcription of gene 3.

AP1 sites and activate transcription (Smeal *et al.*, 1989). V-*fos* differs from c-*fos* in its C-terminal 49 amino acids as a result of a deletion which alters the reading frame of the viral gene (van Beveren *et al.*, 1983). However, both v-*fos* and c-*fos* proteins form specific DNA-binding heterodimers with *jun*, which activate transcription from TPA-responsive elements (Rauscher *et al.*, 1988; Chiu *et al.*, 1988) and both will transform fibroblasts (Miller *et al.*, 1984). Therefore, it appears that it is loss of normal regulation of *fos* protein expression and not the altered C-terminus, which is responsible for a transforming ability of v-*fos*.

Subsequently, a number of other proteins which share the bZip domain present in *jun* and *fos* have been identified (Ryder *et al.*, 1988; Ryder *et al.*, 1989; Hirai *et al.*, 1989; Zerial *et al.*, 1989; Cohen & Curran, 1988) and have been shown to be capable of selectively forming heterodimers (Smeal *et al.*, 1989; Kouzarides & Ziff, 1989; Hai *et al.*, 1989). This gives rise to a considerable repertoire of protein combinations able to bind to the same, or closely related, DNA sequences under different conditions and enables subtle and specific transcriptional regulation of many genes.

### *The* myc *oncogenes*

One of the earliest viral transforming genes to be recognised was that carried by the avian retrovirus MC29, v-*myc*. The protein products of its cellular counterpart, c-*myc*, and the related L- and N-*myc* genes, are expressed in the nucleus (Abrams *et al.*, 1982; Donner *et al.*, 1982) and show a rapid response to growth stimuli (Kelly *et al.*, 1983). Inappropriate expression of different members of the *myc* family, either alone or in conjunction with chromosomal translocation or gene amplification, has been demonstrated in a number of human tumours (Erikson *et al.*, 1983; Cole, 1986; Nau *et al.*, 1985; Schwab *et al.*, 1983). In addition, like *fos*, *myc* can complement *ras* oncogenes in transforming cells (Land *et al.*, 1983). However, the function of the *myc* proteins has remained a mystery, for although they could be shown to bind non-specifically to DNA (Abrams *et al.*, 1982), little more was known. Recently, however, the *myc* proteins were shown to possess the basic/helix-loop-helix (bHLH) motif. This domain is also found in a number of known and putative transcription factors involved in differentiation and tissue-specific gene expression (Davis *et al.*, 1990; Murre *et al.*, 1989*a*, *b*). To the C-terminal side of the bHLH domain, *myc* also has a leucine zipper domain, similar to that present in *fos* and *jun*, which has also been shown to be necessary for the formation of *myc* multimers, but which also may enable interaction with other proteins (Dang *et al.*, 1989). These data suggest strongly that *myc* functions, at least in part, as a transcription factor and that it has the potential for interaction with a wide variety of other proteins.

### c-myb

The oncogene v-*myb*, carried by two chicken retroviruses, avian myeloblastosis virus and E26, is a truncated version of the cellular proto-oncogene c-*myb* (Gonda & Bishop, 1983; Klempnauer *et al.*, 1983) which encodes a nuclear protein of MW 75–80 kD (Klempnauer *et al.*, 1983, 1984, 1986) expressed in immature, but not in differentiated haematopoietic cells (Duprey & Boettiger, 1985). When chicken bone marrow cells are transformed with v-*myb* their differentiation is blocked and they have the phenotype of myeloid precursors (Gazzolo *et al.*, 1979), whilst overexpression of c-*myb* prevents the induction of differentiation of cultured erythroleukaemia cells (McMahon *et al.*, 1988). Both v- and c-*myb* bind specifically to the sequence PyAACG/TG (Py = pyrimidine) and activate transcription from reporter genes (i.e. foreign genes with assayable products) linked in *cis* to this motif (Howe *et al.*, 1990; Weston & Bishop, 1989; Sakura *et al.*, 1989). A cellular gene, *mim*1, encoding a promyelocyte specific secretory protein, which is activated by *myb*, has been identified (Ness *et al.*, 1989). Very recently, it has been shown that phosphorylation of certain serine residues in c-*myb*, by casein kinase II, prevents it from binding to DNA. The region of c-*myb* containing these residues is deleted from v-*myb* and activated c-*myb* genes (Luscher *et al.*, 1990). Transformation is probably the result of expression of a *myb* protein which cannot be prevented from binding to DNA and which thus inappropriately activates transcription from genes responsible for cell growth.

### *Other nuclear oncogenes*

At least five other known viral oncogenes encode proteins which are, or are extremely likely to be, deregulated transcription factors. The best characterised of these is v-*ErbA*, one of the transforming genes of the avian erythroblastosis virus, which encodes a truncated thyroid hormone receptor (Sap *et al.*, 1986; Weinberger *et al.*, 1986), a relative of the steroid hormone receptors (see above) (Evans, 1988; Thompson & Evans, 1989). As a result of deletion of part of the C-terminus of the normal receptor, the viral ErbA protein is unable to bind thyroid hormone. It does, however, bind correctly to thyroid hormone-responsive DNA elements, functioning as a constitutive repressor of thyroid hormone responsive genes and competitive antagonist of the normal thyroid hormone receptor/ligand complex (Damm *et al.*, 1989), suggesting that it may transform erythroblasts by blocking thyroid hormone-mediated differentiation.

v-*ets* is a second oncogene present in the genome of avian leukosis virus, E26, which also bears v-*myb* and is necessary for it to induce erythroblastosis (Leprince *et al.*, 1983; Nunn *et al.*, 1983; Nunn & Hunter, 1989). The product of the chicken proto-oncogene, c-*ets*-1, from which it is derived, is a member of a family of nuclear phosphoproteins of short half-life, expressed predominantly by proliferating cells (Fujiwara *et al.*, 1988*a*, *b*; Pognonec *et al.*, 1989). *Ets* and related proteins are also present in insects and other vertebrates (Pribyl *et al.*, 1988; Reddy *et al.*, 1987; Rao *et al.*, 1989) and all share a highly conserved region of basic amino acids at the C-terminus (Watson *et al.*, 1988). c-*ets*-1 binds specifically to a *cis*-acting element in the long terminal repeat (LTR) of the Moloney murine sarcoma virus (Gunther *et al.*, 1990) and to a closely related sequence, PEA3, in the polyoma virus enhancer. It activates transcription from these sequences and, in the polyoma enhancer, functions cooperatively with members of the *fos-jun* family which bind to an adjacent AP1 site (Wasylyk *et al.*, 1990). In addition, juxtaposed AP1 and PEA3 sites are found in the promoter of a number of oncogene-responsive genes (Wasylyk *et al.*, 1989).

The avian retrovirus ReVT, which causes rapidly progressive lymphoid tumours in birds, carries the v-*rel* oncogene (Stephens *et al.*, 1983). Its product is found in the nucleus of some cells and in the cytoplasm of others (Gilmore & Temin, 1988). The proto-oncogene c-*rel* has recently been shown to be related to, and capable of participating in heterotetrameric complexes with one subunit of NFκB, a multifunctional transcription factor, and *dorsal*, a *Drosophila* protein involved in dorsal/ventral axis determination (Kieran *et al.*, 1990; Ghosh *et al.*, 1990). NFκB is produced as a large non-DNA binding precursor and its activity may be regulated partly through the rate of cleavage of this molecule to a smaller form. However sequestration in the cytoplasm by the related protein IκB, which cannot bind to DNA, is probably more important (Bauerle & Baltimore, 1988). It appears that, unlike c-*rel*, v-*rel* cannot activate transcription, but it retains the ability to interact with other related proteins. Thus it may interfere with the normal equilibrium between active and inactive complexes and lead to aberrant transcriptional regulation.

The nuclear oncogenes v-*ski* and v-*maf* are less well characterised than those described above. V-*ski* was isolated from an avian leukosis virus in culture and the protein it encodes has several features which suggest it may be a transcription factor (Stavnezer *et al.*, 1989). The function of the c-*ski* proto-oncogene from which it is derived is unknown, however transformation of quail embryo cells with v-*ski* causes them to undergo myogenic differentiation suggesting a relationship to the MyoD family of myogenic proteins (Davis *et al.*, 1990; Colmenares & Stavnezer, 1989). V-*maf*, which causes naturally occurring fibrosarcomas in fowl, shows regions of structural similarity to the *fos* and *jun* oncogenes having both a leucine zipper motif (Nishizawa *et al.*, 1989) and an adjacent domain of basic amino acids which fits the consensus sequence of the bZip proteins (Vinson *et al.*, 1989). In addition it has stretches of uninterrupted glycine and histidine residues, a single polyglycine sequence being a feature of the leucine zipper transcription factor, C/EBP (Landschulz *et al.*, 1988*b*).

### Abnormalities of transcription factors in human tumours

Whilst the identification and characterisation of oncogenic transcription factors has told us much about the mechanisms of transcriptional control and deregulation, to date only the expression of *myc* proto-oncogenes, whose transcriptional role is still not finally proven, has been shown to be abnormal in human tumours. However, some recent work has demonstrated more clearly the direct role of other transcription factors in human neoplasia (Table I).

### *Pre-B ALL and helix-loop-helix proteins*

The tumour cells of 30% of patients with childhood pre B-Acute Lymphoblastic Leukaemia (pre B-ALL) carry the translocation t(1:19) (q23:p13.3) (Williams *et al.*, 1984). Two groups investigating the translocation breakpoint have shown that a chimæric gene is produced, from which a potentially functional transcription factor is synthesised (Nourse *et al.*, 1990; Kamps *et al.*, 1990). The N-terminal part of the novel protein is contributed by the E2a gene from chromosome 19, encoding the *myc*-related proteins E12 and E47, which normally stimulate constitutive transcription from the kappa immunoglobulin promoter in B-cells (Murre *et al.*, 1989*b*; Moss *et al.*, 1988). The part of the protein retained in the chimæric factor is the potent transcriptional activator domain. The C-terminus, encoded by part of a gene on chromosome 1, normally inactive in pre-B lymphocytes, is the homeodomain of a newly identified factor of unknown function, designated prl. Many of the proteins sharing this structural feature are involved in developmental and tissue-specific regulation of gene expression (Gehring, 1987; Ingraham *et al.*, 1988; Scheidereit *et al.*, 1988; Johnson & Hirsh, 1990). Thus, the result of the pre B-ALL translocation is a protein which may be capable of strongly activating a gene or group of genes involved in the control of normal differentiation and not normally expressed at this stage of lymphocyte ontogeny.

Two additional bHLH proteins, relatives of E12/E47, have been implicated in different forms of acute leukaemia. One, SCL, was identified as the protein encoded by the gene on chromosome 1 involved in the breakpoint of the translocation, t(1:14) (p33;q11.2) in a primitive acute leukaemia capable of both myeloid and lymphoid differentiation. The normal SCL gene is expressed by haematopoietic stem cells and myeloid and T lymphocyte precursors (Begley *et al.*, 1989) whilst an aberrant mRNA, including at its 3′ end part of the T-cell receptor (TCR) $\delta$ chain gene, is produced by tumour cells. The same gene is also involved in the translocation t(1:14) (p32;q11) in a small group of T-cell acute lymphoblastic leukaemias (T-ALL) (Chen *et al.*, 1990). In these malignant cells the SCL gene is fused to the 5′ part of the TCR $\delta$ gene and thus it may be expressed under the control of an inappropriate promoter. The second protein, designated Lyl-1, is closely related to SCL and is expressed in another subset of T-ALLs, in a truncated form, as the result of a t(7:19) translocation which juxtaposes its gene to that of the TCR $\beta$ chain (Mellentin *et al.*, 1989). Further study of these proteins should give valuable insight into the role of transcription in normal haematopoietic development and in neoplasia.

### *Wilms' tumour and the zinc finger proteins*

Gross cytogenetic deletions and rearrangements affecting chromosome 11 (band p13) are common finding in hereditary and some sporadic Wilm's tumours. The gene inactivated by these changes encodes a protein (the WT protein) with four 'zinc fingers' near to its C-terminus suggesting that it is a transcription factor (Call *et al.*, 1990; Gessler *et al.*, 1990). Its N-terminus is rich in both proline and glutamine residues, amino acids occurring frequently in the activation domains of some other transcription factors (Mermod *et al.*, 1989; Tanaka & Herr, 1990). The gene is strongly expressed in the embryonic kidney and its product appears to be required for the switch from mesodermal to epithelial differentiation which occurs in the developing urogenital sysem (Pritchard-Jones *et al.*, 1990). The mechanism by which homologous deletion or rearrangement of the WT gene leads to the uncontrolled proliferation of nephroblasts is unknown, but one might speculate that the normal WT protein stimulates their differentiation into nephrons and its inactivation allows continued growth and division. This is supported by the inability of nephroblastoma cells transfected with a normal chromosome 11, to form tumours, but more definitive work is awaited (Weissman *et al.*, 1987).

In acute promyelocytic leukaemia (APL; FABM3), tumour cells carry the translocation t(15:17) (q22; q11.2–q12) in approaching 100% of cases (Sheer *et al.*, 1984). The chromosome 17 breakpoint has been characterised in a number of cases and shown to fall in the first intron of the retinoic acid receptor $\alpha$ (RARA) gene (Borrow *et al.*, 1990). The product of this gene is related to the steroid hormone receptors, and as a result of the translocation, the first exon, which encodes the transcription activation domain, is separated from the DNA-binding and ligand-binding domains, which become part of the derivative chromosome 15. The gene to which they are attached awaits characterisation, but as with pre-B ALL the resultant fusion protein is likely to be of great importance in understanding the behaviour of the tumour cells and probably explains the response of APL to retinoic acid.

Other zinc finger proteins have also been implicated in neoplasia. The gene GLI, on chromosome 12, is amplified in a proportion of human malignant gliomas (Kinzler *et al.*, 1987). Evidence suggests that the GLI protein may be involved in normal development as it is most closely related to the 'Kruppel' zinc finger proteins (Kinzler *et al.*, 1988) involved in *Drosophila* segmentation (Schuh *et al.*, 1986; Chowdhury *et al.*, 1987) and is produced by embryonal carcinoma cells but not by adult tissues.

The translocation, t(11:14) (p15;q11), present in a group of T-ALL activates the gene, *ttg*, (McGuire *et al.*, 1989; Boehm *et al.*, 1988), which encodes a zinc finger protein normally expressed in the developing nervous system and in certain regions of the mature brain (Greenberg *et al.*, 1990). It is likely that that proteins described above are not the only developmentally regulated transcription factors with a role in transformation.

### Future prospects

In addition to yielding a greater understanding of the malignant process, the study of transcription may also provide advances in the diagnosis, assessment of prognosis and treatment of cancer. Whilst the inappropriate expression or repression of various genes may underlie the behaviour and histological appearance of a malignant tumour, in reaching a diagnosis the pathologist relies, to a great extent, upon the persistence of some features of differentiation in the tumour cells. For example, the identification of cytokeratins in epithelial tumours; of melanin and neuroendocrine markers in melanomas; of desmin, myoglobin and the cross striations produced by the contractile machinery of skeletal muscle in rhabdomyosarcomas; and of leucocyte common antigen in lymphomas frequently aids assessment. Unfortunately, these

**Table I** Some abnormalities of transcription factors in human tumours

| *Tumour* | *%* | *Genes* | *Mechanism* | *Result* |
|---|---|---|---|---|
| Pre-B-ALL | 30 | E2a *prl* | t(1:19) | Chimaeric protein[1,2] |
| Stem cell Leukaemia | – | SCL/*tal* TCRδ | t(1:14) | Abnormal mRNA[3] |
| T-ALL | 2–20 | SCL/*tal* TCRδ | t(1:14) | Inappropriate expression[4] |
| T-ALL | ⩽10 | *lyl*-1 TCRβ | t(7:19) | Truncated mRNA[5] |
| T-ALL | ⩽10 | ttg TCRδ | t(11:14) | Inappropriate expression[6] |
| T-ALL | ⩽10 | c-*myc* TCRα/δ | t(8:14) | Inappropriate expression[7] |
| Wilms' Tumour | | WT | deletion/mutation | Inactivation[8,9] |
| APL | c100 | RARA ? | t(15:17) | Chimaeric protein[10] |
| Malignant glioma | 4 | *gli* | Amplification | Overexpression[11,12] |
| Burkitt's lymphoma | 75 16 9 | c-*myc* IgH/κ/λ | t(8:14) t(8:22) t(2:8) | Inappropriate expression[13,14] |
| Various | – | c-*myc* | Amplification | Overexpression |

Refs: [1]Nourse *et al.*, 1990; [2]Kamps *et al.*, 1990; [3]Begley *et al.*, 1989; [4]Chen *et al.*, 1990; [5]Mellentin *et al.*, 1989; [6]McGuire *et al.*, 1989; [7]Shima *et al.*, 1986; [8]Call *et al.*, 1990; [9]Gessler *et al.*, 1990; [10]Borrow *et al.*, 1990; [11]Kinzler *et al.*, 1987; [12]Wong *et al.*, 1987; [13]Erikson *et al.*, 1983; [14]Nishikura *et al.*, 1983.

markers are produced in different amounts by individual tumours of the same type and in a proportion no evidence of differentiation can be identified, making classification and therefore rational treatment difficult.

The identification of tissue-specific transcription factors in cancer cells could offer a considerable advance in tumour diagnosis. As described above, it is likely that transcription factors are responsible for determining the line of differentiation undertaken by a cell and such factors must be expressed prior to the genes which they regulate. Already a number of proteins controlling muscle-specific gene expression, which cause differentiation of fibroblasts into skeletal muscle have been cloned (Davis *et al.*, 1990; Miner & Wold, 1990; Braun *et al.*, 1989; Braun *et al.*, 1990; Wright *et al.*, 1989; Rhodes & Konieckny, 1989), whilst factors binding to HMW keratin promoters have been identified in keratinocytes (Lersch *et al.*, 1989; Jiang *et al.*, 1990). In the future the demonstration of tissue-specific transcription factors within otherwise undifferentiated tumour cells may enable a correct diagnosis to be reached and thus appropriate chemotherapy to be given.

With regard to prognosis and treatment, assessment and manipulation of transcription factors is already commonplace in many centres; the oestrogen and progesterone receptor status of breast carcinomas is a routinely applied and valuable, prognostc indicator (DeSombre *et al.*, 1979; Maynard *et al.*, 1978) and administration of tamoxifen, a competitive oestrogen antagonist, is a standard treatment. When tamoxifen binds to the oestrogen receptor, dimerisation and DNA binding occur normally, but transcriptional activation is inhibited (Kumar & Chambon, 1988). Thus oestrogen-mediated stimulation of growth-related genes is prevented. A similar situation is likely to result from the administration of anti-androgens, such as cyprotersone acetate, to patients with prostatic carcinoma. The treatment of lymphoma with corticosteroids may act via the glucocorticoid receptor either to suppress transcription from genes involved in cell division or to stimulate differentiation (Beato, 1989; Thompson, 1989). In the future, as a greater understanding of the transcriptional machinery is gained, it is likely firstly, that other transcription factors will be identified whose production by a tumour will correlate with a better or a worse prognosis and secondly, that drugs will be developed which can interact with the transcriptional machinery to repress cell growth and division or stimulate differentiation of tumour cells. Whilst this is currently only speculation, transcription-related research should soon yield major dividends for cancer patients.

### References

ABRAMS, H.D., ROHRSCHNEIDER, L.R. & EISENMAN, R.N. (1982). Nuclear location of the putative transforming protein of avian myelocytomatosis virus. *Cell*, **29**, 427.

AKAM, M. (1987). The molecular basis for metameric pattern in the Drosophila embryo. *Development*, **101**, 1.

ANGEL, P., ALLEGRETTO, E.A., OKINO, S.T. & 4 others (1988). Oncogene jun encodes a sequence-specific trans-activator similar to AP-1. *Nature*, **332**, 166.

ANGEL, P., IMAGURA, M., CHIU, R. & 6 others (1987). Phorbol ester-inducible genes contain a common cis element recognised by a TPA-modulated transacting factor. *Cell*, **49**, 729.

BAEUERLE, P.A. & BALTIMORE, D. (1988). I-κB: a specific inhibitor of the NF-κB transcription factor. *Science*, **242**, 540.

BAICHWAL, V.R. & TJIAN, R. (1990). Control of c-*Jun* activity by integration of a cell-specific inhibitor with regulatory domain δ: differences between v- and c-*Jun*. *Cell*, **63**, 815.

BATTEY, J., MOULDING, C., TAUB, R. & 5 others (1983). The human c-*myc* oncogene: structural consequences of translocation into the IgH locus in Burkitt lymphoma. *Cell*, **34**, 779.

BEATO, M. (1989). Gene regulation by steroid hormones. *Cell*, **56**, 335.

BEGLEY, C.G., APLAN, P.D., DENNING, S.M., HAYNES, B.F., WALDMANN, T.A. & KIRSCH, I.R. (1989). The gene SCL is expressed during early hematopoiesis and encodes a differentiation-related DNA-binding motif. *Proc. Natl Acad. Sci. USA*, **86**, 10128.

BENBROOK, D.M. & JONES, N.C. (1990). Heterodimer formation between CREB and JUN proteins. *Oncogene*, **5**, 295.

BENEZRA, R., DAVIS, R.L., LOCKSHON, D., TURNER, D.L. & WEINTRAUB, H. (1990). The protein Id: a negative regulator of helix-loop-helix DNA binding proteins. *Cell*, **61**, 49.

BERNARDS, R. (1987). Suppression of MHC gene expression in cancer cells. *Trends Genet.*, **3**, 298.

BOEHM, T., BAER, R., LAVENIR, I. & 4 others (1988). The mechanism of chromosomal translocation t(11:14) involving the T-cell receptor Cδ locus on human chromosome 14q11 and a transcribed region of chromosome 11p15. *EMBO J.*, **7**, 385.

BORROW, J., GODDARD, A.D., SHEER, D. & SOLOMON, E. (1990). Molecular analysis of acute promyelocytic leukemia breakpoint cluster region on chromosome 17. *Science*, **249**, 1577.

BOS, T.J., BOHMANN, D., TSUCHIE, H., TJIAN, R. & VOGT, P.K. (1988). v-*jun* encodes a nuclear protein with enhancer binding properties of AP1. *Cell*, **52**, 705.

BRAUN, T., BOBER, E., WINTER, B., ROSENTHAL, N. & ARNOLD, H.H. (1990). Myf-6, a new member of the human gene family of myogenic determination factors: evidence for a gene cluster on chromosome 12. *EMBO J.*, **9**, 821.

BRAUN, T., BUCSCHHAUSEN-DENKER, G., BOBER, E., TANNICH, E. & ARNOLD, H.H. (1989). A novel human muscle factor related to but distinct from MyoD1 induces myogenic conversion of 10T1/2 fibroblasts. *EMBO J.*, **8**, 701.

BROWN, R.S., SANDER, C. & ARGOS, P. (1985). The primary structure of transcription factor TFIIIA has 12 consecutive repeats. *FEBS Lett.*, **186**, 271.

CALL, K.M., GLASER, T., ITO, C.Y. & 9 others (1990). Isolation and characterisation of a zinc-finger polypeptide gene at the human chromosome 11 Wilm's tumour locus. *Cell*, **60**, 509.

CHEN, Q., CHENG, J.-T., TSAI, L-H. & 7 others (1990). The tal gene undergoes chromosome translocation in T cell leukaemia and potentially encodes a helix-loop-helix protein. *EMBO J.*, **9**, 415.

CHIU, R., BOYLE, W.J., MEEK, J., SMEAL, T., HUNTER, T. & KARIN, M. (1988). The c-*fos* protein interacts with c-*jun*/AP-1 to stimulate transcription of AP-1 responsive genes. *Cell*, **54**, 541.

CHODOSH, L.A., BALDWIN, A.S., CARTHEW, R.W. & SHARP, P.A. (1988). Human CCAAT-binding proteins have heterologous subunits. *Cell*, **53**, 11.

CHOWDHURY, K., DEUTSCH, U. & GRUSS, P. (1987). A multigene family encoding several 'finger' structures is present and differentially active in mammalian genomes. *Cell*, **48**, 771.

COHEN, D.M. & CURRAN, T. (1988). *fra*-1: a serum-inducible, cellular immedate-early gene that encodes a *fos*-related antigen. *Mol. Cell Biol.*, **8**, 2063.

COLE, M.D. (1986). The *myc* oncogene: its role in transformation and differentiation. *Ann. Rev. Genet.*, **20**, 361.

COLMENARES, C. & STAVNEZER, E. (1989). The *ski* oncogene induces muscle differentiation in quail embryo cells. *Cell*, **59**, 293.

COX, P.M., TEMPERLEY, S.M., KUMAR, H. & GODING, C. (1988). A distinct octamer binding protein present in malignant melanoma cells. *Nucleic Acids Res.*, **16**, 11047.

CURRAN, T., MILLER, A.D., ZOKAS, L. & VERMA, I.M. (1984). Viral and cellular fos proteins: a comparative analysis. *Cell*, **36**, 259.

DAMM, K., THOMPSON, C.C. & EVANS, R.M. (1989). Protein encoded by v-*Erb* A functions as a thyroid hormone receptor antagonist. *Nature*, **339**, 593.

DANG, C.V., MCGUIRE, M., BUCKMIRE, M. & LEE, W.M.F. (1989). Involvement of the leucine zipper region in the oligomerization and transforming activity of the c-*myc* protein. *Nature*, **337**, 664.

DAVIS, R.L., CHENG, P.-F., LASSAR, A.B. & WEINTRAUB, H. (1990). The MyoD DNA-binding domain contains a recognition code for muscle-specific gene activation. *Cell*, **60**, 733.

DESOMBRE, E.R., CARBONE, P.P. & JENSEN, E.V. (1979). Steroid receptors in breast cancer. *N. Engl. J. Med.*, **301**, 1011.

DIAKUN, G.P., FAIRALL, L. & KLUG, A. (1986). EXAFS study of the zinc-binding sites in the protein transcription factor IIIA. *Nature*, **324**, 698.

DISTEL, R.J., RO, H.-S., ROSEN, B.S., GROVES, D.L. & SPIEGELMAN, B.M. (1987). Nucleoprotein complexes that regulate gene expression in adipocyte differentiation: direct participation of c-*fos*. *Cell*, **49**, 835.

DONNER, P., GREISER-WILKE, I. & MOELLING, K. (1982). Nuclear localization and DNA binding of the transforming gene product of avian myelocytomatosis virus. *Nature*, **296**, 262.

DORN, A., BOLLEKENS, J., STAU, B.A., BENOIST, C. & MATHIS, D. (1987). A multiplicity of CCAAT box binding proteins. *Cell*, **50**, 863.

DUPREY, S.P. & BOETTIGER, D. (1985). Developmental regulation of c-myb in normal myeloid progenitor cells. *Proc. Natl Acad. Sci. USA*, **82**, 6937.

DYNAN, W.S. & TJIAN, R. (1985). Control of eukaryotic messenger RNA synthesis by sequence-specific DNA-binding proteins. *Nature*, **316**, 774.

ELLIS, H.M., SPANN, D.R. & POSAKONY, J.W. (1990). Extramacroachaete: a negative regulator of sensory organ development in *Drosophila*, defines a new class of helix-loop-helix proteins. *Cell*, **61**, 27.

ERIKSON, J., AR-RUSHDI, A., DRWINGA, H.L., NOWELL, P.C. & CROCE, C.M. (1983). Transcriptional activation of the translocated c-*myc* oncogene in Burkitt lymphoma. *Proc. Natl Acad. Sci. USA*, **80**, 820.

EVANS, R.M. (1988). The steroid and thyroid hormone receptor superfamily. *Science*, **240**, 889.

FINNEY, M., RUVKUN, G. & HORVITZ, H.R. (1988). The *C. elegans* cell lineage and differentiation gene unc86 encodes a protein containing a homeodomain and extended sequence similarity to mammalian transcription factors. *Cell*, **55**, 757.

FUJIWARA, S., FISHER, R.J., SETH, A. & 4 others (1988). Characterisation and localisation of the products of the human homologs of the v-*ets* oncogene. *Oncogene*, **2**, 99.

FUJIWARA, S., FISHER, R.J., BHAT, N.K., ESPINA, S. & PAPAS, T.S. (1988). A short lived nuclear phosphoprotein encoded by the human *ets*-2 proto-oncogene is stabilized by activation of protein kinase C. *Mol. Cell. Biol.*, **8**, 4700.

GARRELL, J. & MODOLELL, J. (1990). The *Drosophila* extramacroachaete locus, an antagonist of proneural genes that, like these genes, encodes a helix-loop-helix protein. *Cell*, **61**, 39.

GAZZOLO, L., MOSCOVICI, C. & MOSCOVICI, M.G. (1979). Response of haemopoietic cells to avian acute leukaemia viruses: effects on the differentiation of the target cells. *Cell*, **16**, 627.

GEHRING, W.J. (1987). Homeoboxes in the study of development. *Science*, **236**, 1245.

GESSLER, M., POUSTKA, A., CAVENEE, W., NEVE, R.L., ORKIN, R.L. & BRUNS, G.A.P. (1990). Homozygous deletion in Wilm's tumours of a zinc-finger gene identified by chromosome jumping. *Nature*, **343**, 774.

GHOSH, S., GIFFORD, A.M., RIVIERE, L.R., TEMPST, P., NOLAN, G.P. & BALITMORE, D. (1990). Cloning of the p50 subunit of NF-κB: homology to *rel* and dorsal. *Cell*, **62**, 1019.

GILMORE, T.D. & TEMIN, H.M. (1988). V-*rel* oncoproteins in the nucleus and in the cytoplasm transform chicken spleen cells. *J. Virol.*, **62**, 703.

GONDA, T.J. & BISHOP, J.M. (1983). Structure and transcription of the cellular homolog (c-*myb*) of the avian myeloblastosis virus transforming gene (v-*myb*). *J. Virol.*, **46**, 212.

GRAHAM, A., PAPALOPULU, N. & KRUMLAUF, R. (1989). The murine and *Drosophila* homeobox gene complexes have common features of organisation and expression. *Cell*, **57**, 367.

GREEN, S. & CHAMBON, P. (1987). Oestradiol induction of a glucocorticoid-responsive gene by a chimeric receptor. *Nature*, **325**, 75.

GREEN, S. & CHAMBON, P. (1988). Nuclear receptors enhance our understanding of transcription regulation. *Trends Genet.*, **4**, 309.

GREENBERG, J.M., BOEHM, T., SOFRONIEW, M.V. & 6 others (1990). Segmental and developmental regulation of a presumptive T-cell oncogene in the central nervous system. *Nature*, **344**, 158.

GREGOR, P.D., SAWADOGO, M. & ROEDER, R.G. (1990). The adenovirus major late transcription factor USF is a member of the helix-loop-helix group of regulatory proteins and binds to DNA as a dimer. *Genes. Dev.*, **4**, 1730.

GUNTHER, C.V., NYE, J.A., BRYNER, R.S. & GRAVES, B.J. (1990). Sequence-specific DNA binding of the proto-oncogene *ets*-1 defines a transcriptional activator sequence within the long terminal repeat of the Moloney murine sarcoma virus. *Genes Dev.*, **4**, 667.

HAI, T., LIU, F., COUKOS, W.J. & GREEN, M.R. (1989). Transcription factor ATF cDNA clones: an extensive family of leucine zipper proteins able to selectively form DNA-binding heterodimers. *Genes. Dev.*, **3**, 2083.

HERR, W., STURM, R.A., CLERC, R.G. & 8 others (1988). The POU domain: a large conserved region in the mammalian *pit*-1, *oct*-1, *oct*-2 and *Caenorhabditis elegans unc*-86 gene products. *Genes Dev.*, **2**, 1513.

HILL, D.E., HOPE, I.A., MACKE, J.P. & STRUHL, K. (1986). Saturation mutagenesis of the yeast his 3 regulatory site: requirements for transcriptional induction and for binding by GCN4 activator protein. *Science*, **234**, 451.

HIRAI, S.-I., RYSECK, R.-P., MECHTA, F., BRAVO, R. & YANIV, M. (1989). Characterisation of *jun* D: a new member of the *jun* proto-oncogene family. *EMBO J*, **8**, 1433.

HOLLAND, P.W.H. & HOGAN, B.L.M. (1988). Expression of homeobox genes during mouse development: a review. *Genes. Dev.*, **2**, 773.

HOWE, K.M., REAKES, C.F.L. & WATSON, R.J. (1990). Characterisation of the sequence-specific interaction of mouse c-*myb* protein with DNA. *EMBO J.*, **9**, 161.

HU, Y.-F., LUSCHER, B., ADMON, A., MERMOD, N. & TJIAN, R. (1990). Transcription factor AP4 contains multiple dimerisation domains that regulate dimer specificity. *Genes Dev.*, **4**, 1741.

INGRAHAM, H.A, CHEN, R., MANGALAM, H.J. & 6 others (1988). A tissue-specific transcription factor containing a homeodomain specifies a pituitary phenotype. *Cell*, **55**, 519.

JIANG, C.-K., EPSTEIN, H.S., TOMIC, M., FREEDBURG, I.M. & BLUMENBERG, M. (1990). Epithelial-specific keratin gene expression: identification of a 300-base-pair controlling fragment. *Nucleic Acids Res.*, **18**, 247.

JOHNSON, P.F. & MCKNIGHT, S.L. (1989). Eukaryotic transcriptional regulatory proteins. *Annu. Rev. Biochem.*, **58**, 799.

JOHNSON, W.A. & HIRSH, J. (1990). Binding of a *Drosophila* POU-domain protein to a sequence element regulating gene expression in specific dopaminergic neurons. *Nature*, **343**, 467.

KADONAGA, J.T., CARNER, K.R., MASIARZ, F.R. & TJIAN, R. (1987). Isolation of cDNA encoding transcription factor SP1 and functional analysis of the DNA binding domain. *Cell*, **51**, 1079.

KAMPS, M.P., MURRE, C., SUN, X.-H. & BALTIMORE, D. (1990). A new homeobox gene contributes the DNA binding domain of the t(1:19) translocation protein in preB-ALL. *Cell*, **60**, 547.

KELLY, K., COCHRAN, B.H., STILES, C.D. & LEDER, P. (1983). Cell-specific regulation of the c-myc gene by lymphocyte mitogens and platelet-derived growth factor. *Cell*, **35**, 603.

KIERAN, M., BLANK, V., LOGEAT, F. & 7 others (1990). The DNA-binding subunit of NF-κB is identical to factor KBF1 and homologous to the *rel* oncogene product. *Cell*, **62**, 1007.

KINZLER, K.W., BIGNER, S.H., BIGNER, D.D. & 5 others (1987). Identification of an amplified, highly expressed gene in a human glioma. *Science*, **236**, 70.

KINZLER, K.W., RUPPERT, J.M., BIGNER, S.H. & VOGELSTEIN, B. (1988). The GLI gene is a member of the Kruppel family of zinc finger proteins. *Nature*, **332**, 371.

KISSINGER, C.R., LIU, B., MARTIN-BLANCO, E., KORNBERG, T.B. & PABO, C.O. (1990). Crystal structure of an engrailed homeodomain-DNA complex at 2.8Å resolution: a framework for understanding homeodomain-DNA interactions. *Cell*, **63**, 579.

KLEMPNAUER, K.-H., BONIFER, C. & SIPPEL, A.E. (1986). Identification and characterisation of the protein encoded by the human c-*myb* proto-oncogene. *EMBO J.*, **5**, 1903.

KLEMPNAUER, K.-H., RAMSAY, G., BISHOP, J.M. & 4 others (1983). The product of the retroviral transforming gene, v-*myb*, is a truncated version of the protein encoded by the cellular oncogene c-*myb*. *Cell*, **33**, 345.

KLEMPNAUER, K.-H., SYMONDS, G., EVAN, G.I. & BISHOP, J.M. (1984). Subcellular localisation of proteins encoded by oncogenes of avian myeloblastosis virus and avian leukaemia virus E26 and the chicken c-*myb* gene. *Cell*, **37**, 537.

KOUZARIDES, T. & ZIFF, E. (1989). Leucine zippers of *fos*, *jun* and GCN4 dictate dimerisation specificity and thereby control DNA binding. *Nature*, **340**, 568.

KUMAR, V. & CHAMBON, P. (1988). The estrogen receptor binds tightly to its responsive element as a ligand-induced homodimer. *Cell*, **55**, 145.

LAND, H., PARADA, L.F. & WEINBERG, R.A. (1983). Tumorigenic conversion of primary embryo fibroblasts requires at least two cooperating oncogenes. *Nature*, **304**, 596.

LANDSCHULZ, W.H., JOHNSON, P.F. & MCKNIGHT, S.L. (1988*a*). The leucine zipper: a hypothetical structure common to a new class of DNA binding proteins. *Science*, **240**, 1759.

LANDSCHULZ, W.H., JOHNSON, P.F., ADASHI, E.Y., GRAVES, B.J. & MCKNIGHT, S.L. (1988*b*). Isolation of a recombinant copy of the gene encoding C/EBP. *Genes Dev.*, **2**, 786.

LAUGHON, A. & SCOTT, M.P. (1984). Sequence of a *Drosophila* segmentation gene: protein structure homology with DNA-binding proteins. *Nature*, **310**, 25.

LEE, W., MITCHELL, P. & TJIAN, R. (1987). Purified transcription factor AP-1 interacts with TPA-inducible enhancer elements. *Cell*, **49**, 741.

LENARDO, M., PIERCE, J.W. & BALTIMORE, D. (1987). Protein-binding sites in Ig gene enhancers determine transcriptional activity and inducibility. *Science*, **236**, 1573.

LEPRINCE, D., GEGONNE, A., COLL, C. & 4 others (1983). A putative second cell-derived oncogene of the avian leukaemia retrovirus E26. *Nature*, **306**, 395.

LERSCH, R., STELMACH, V., STOCKS, C., GUIDICE, G. & FUCHS, E. (1989). Isolation, sequence and expression of a human keratin K5 gene: transcriptional regulation of keratins and insights into pair-wise control. *Mol. Cell. Biol.*, **9**, 3685.

LUSCHER, B., CHRISTENSON, E., LITCHFIELD, D.W., KREBS, E.G. & EISENMAN, R.N. (1990). *Myb* DNA binding is inhibited by phosphorylation at a site deleted during oncogenic activation. *Nature*, **344**, 517.

MAKI, Y., BOS, T.J., DAVIS, C., STARBUCK, M. & VOGT, P.K. (1987). Avian sarcoma virus 17 carries the *jun* oncogene. *Proc. Natl Acad. Sci USA*, **84**, 8248.

MANIATIS, T., GOODBOURN, S. & FISCHER, J.A. (1987). Regulation of inducible and tissue-specific gene expression. *Science*, **236**, 1237.

MANLEY, J.L. & LEVINE, M.S. (1985). The homeobox and mammalian development. *Cell*, **43**, 1.

MARTIN, G.S. (1970). Rous sarcoma viruses: a function required for the maintenance of the transformed state. *Nature*, **227**, 1021.

MAYNARD, P.V., BLAMCY, R.W., ELSTON, C.W., HAYBITTLE, J.L. & GRIFFITHS, K. (1978). Estrogen receptor assay in primary breast cancer and early recurrence. *Cancer Res.*, **32**, 4292.

MCDONALD, N.J., KUHL, D., MAGUIRE, D. & 9 others (1990). Different pathways mediate virus-inducibility of the human IFN-α1 and IFN-β genes. *Cell*, **60**, 767.

MCGUIRE, E.A., HOCKETT, R.D., POLLOCK, K.M., BARTHOLDI, M.F., O'BRIEN, S.J. & KORSMEYER, S.J. (1989). The t(11:14)(p15;q11) in a T-cell acute lymphoblastic leukaemia cell line activates multiple transcripts, including *ttg*-1, a gene encoding a potential zinc finger protein. *Mol. Cell. Biol.*, **9**, 2124.

MCKNIGHT, S. & TJIAN, R. (1986). Transcriptional selectivity of viral genes in mammalian cells. *Cell*, **46**, 795.

MCMAHON, J., HOWE, K.M. & WATSON, R.J. (1988). The induction of Friend erythroleukaemia differentiation is markedly affected by expression of a transfected c-*myb* cDNA. *Oncogene*, **3**, 717.

MELLENTIN, J.D., SMITH, S.D. & CLEARY, M.D. (1989). *lyl*-1, a novel gene altered by chromosomal translocation in T cell acute leukaemia, codes for a protein with a helix-loop-helix DNA binding motif. *Cell*, **58**, 77.

MELLON, P.L., CLEGG, C.H., CORREL, L.A. & MCKNIGHT, G.S. (1989). Regulation of transcription by cyclic AMP-dependent protein kinase. *Proc. Natl Acad. Sci USA*, **86**, 4887.

MERMOD, N., O'NEILL, E.A., KELLY, T.J. & TJIAN, R. (1989). The proline-rich transcriptional activator of CTF/NF1 is distinct from the replication and DNA binding domain. *Cell*, **58**, 741.

MILLER, A.D., CURRAN, T. & VERMA, I.M. (1984). c-*fos* protein can induce cellular transformation: a novel mechanism of activation of a cellular oncogene. *Cell*, **36**, 51.

MILLER, J., MCLAGHLAN, A.D. & KLUG, A. (1985). Repetitive zinc-binding domains in the protein transcription factor TFIIIA from *Xenopus* oocytes. *EMBO J.*, **4**, 1609.

MINER, J.H. & WOLD, B. (1990). Herculin, a fourth member of the MyoD family of myogenic regulatory genes. *Proc. Natl Acad. Sci. USA*, **87**, 1089.

MIYAMOTO, M., FUJITA, T., KIMURA, Y. & 5 others (1988). Regulated expression of a gene encoding a nuclear factor, IRF-1, that specifically binds to IFNβ gene regulatory elements. *Cell*, **54**, 903.

MOSS, L.G., MOSS, J.B. & RUTTER, W.J. (1988). Systemic binding analysis of the insulin gene transcriptional control region: insulin and immunoglobulin enhancers utilize similar transactivators. *Mol. Cell. Biol.*, **8**, 2620.

MURRE, C., MCCAW, P.S. & BALTIMORE, D. (1989). A new DNA-binding and dimerisation motif in immunoglobulin enhancer-binding, daughterless, MyoD and *myc* proteins. *Cell*, **56**, 777.

MURRE, C., MCCAW, P.S., VAESSIN, H. & 9 others (1989). Interactions between heterologous helix-loop-helix proteins generate complexes that bind specifically to a common DNA sequence. *Cell*, **58**, 537.

NAU, M.M., BROOKS, B.J., BATTEY, J. & 7 others (1985). L-myc, a new *myc*-related gene amplified and expressed in human small cell lung cancer. *Nature*, **318**, 69.

NESS, S.A., MARKNELL, A. & GRAF, T. (1989). The v-*myb* oncogene product binds to and activates the promyelocyte-specific *mim*1 gene. *Cell*, **59**, 1115.

NISHIZAWA, M., KATAOKA, K., GOT, O.N., FUJIWARA, K.T. & KAWAI, S. (1989). v-*maf*, a viral oncogene that encodes a 'leucine zipper' motif. *Proc. Natl Acad. Sci. USA*, **86**, 7711.

NOURSE, J., MELLENTIN, J.D., GALILI, N. & 4 others (1990). Chromosomal translocation t(1:19) results in synthesis of a homeobox fusion mRNA that codes for a potential chimeric transcription factor. *Cell*, **60**, 535.

NUNN, M.F. & HUNTER, T. (1989). The *ets* sequence is required for induction of erythroblastosis in chickens by avian retrovirus E26. *J. Virol.*, **63**, 398.

NUNN, M.F., SEEBURG, P.H., MOSCOVICI, C. & DUESBURG, P.H. (1986). Tripartite structure of the avian erythroblastosis virus E26 transforming gene. *Nature*, **306**, 391.

OGAWA, N. & OSHIMA, Y. (1990). Functional domains of a positive regulatory protein, PHO4, for transcriptional control of the phosphatase regulon in *Saccharomyces cerevisiae*. *Mol. Cell. Biol.*, **10**, 2224.

O'HARE, P., GODING, C. & HAIGH, A. (1988). Direct combinatorial interaction between a *Herpes simplex* virus regulatory protein and a cellular octamer-binding protein mediates specific induction of virus immediate-early gene expression. *EMBO J.*, **7**, 4231.

O'HARE, P. & GODING, C.R. (1988). *Herpes simplex* virus regulatory elements and the immunoglobulin octamer domain bind a common factor and are both targets for virion transactivation. *Cell*, **52**, 435.

PABO, C.O. & SAUER, R.T. (1984). Protein-DNA recognition. *Annu. Rev. Biochem.*, **53**, 293.

PAGE, D.C., MOSHER, R., SIMPSON, E.M. & 6 others (1987). The sex determining region of the human Y chromosome encodes a finger protein. *Cell*, **51**, 1091.

PAWSON, T. (1987). Transcription factors as oncogenes. *Trends Genet.*, **3**, 333.

POGNONEC, P., BOULUKOS, K.E. & GHYSDAEL, J. (1989). The c-*ets*-1 protein is chromatin associated and binds to DNA. *Oncogene*, **4**, 691.

PRIBYL, L.J., WATSON, D.K., MCWILLIAMS, M.J., ASCIONE, R. & PAPAS, T.S. (1988). The *Drosophila ets*-2 gene: molecular structure, chromosomal localization, and developmental expression. *Dev. Biol.*, **127**, 45.

PRITCHARD-JONES, K., FLEMING, S., DAVIDSON, D. & 9 others (1990). The candidate Wilms' tumour gene is involved in genitourinary development. *Nature*, **346**, 194.

PTASHNE, M. (1986). Gene regulation by proteins acting nearby and at a distance. *Nature*, **322**, 697.

RAO, V.N., HUEBNER, K., ISOBE, M. AR-RUSHDI, A., CROCE, C.M. & REDDY, E.S.P. (1989). *elk*, tissue-specific *ets*-related genes on chromosomes X and 14 near translocation breakpoints. *Science*, **244**, 66.

RAUSCHER, F.J., COHEN, D.R., CURRAN, T. & 5 others (1988). *Fos*-associated protein p39 is the product of the *jun* proto-oncogene. *Science*, **240**, 1010.

RAUSCHER, F.J., SAMBUCETTI, L.C., CURRAN, T., DISTEL, R.J. & SPIEGELMAN, B.M. (1988). Common DNA binding site for *fos* protein complexes and transcription factor AP-1. *Cell*, **52**, 471.

RAYMONDJEAN, M., CEREGHINI, S. & YANIV, M. (1988). Several distinct 'CCAAT' box binding proteins coexist in eukaryotic cells. *Proc. Natl Acad. Sci. USA*, **85**, 757.

REDDY, E.S.P., RAO, V.N. & PAPAS, T.S. (1987). The *erg* gene: a human gene related to the *ets* gene. *Proc. Natl Acad. Sci. USA*, **84**, 6131.

RHODES, S.J. & KONIECKNY, S.F. (1989). Identification of MRF4: a new member of the muscle regulatory factor gene family. *Genes Dev.*, **3**, 2050.

ROBERTS, A.B. & SPORN, M.B. (1985). Growth factors and malignancy. *Cancer Surv.*, **4**, 4.

RYDER, K., LANAHAN, A., PEREZ-ALBUERNE, E. & NATHANS, D. (1989). Jun D: a third member of the *jun* gene family. *Proc. Natl Acad. Sci. USA*, **86**, 1500.

RYDER, K., LAU, L.F. & NATHANS, D. (1988). A gene activated by growth factors is related to the oncogene v-*jun*. *Proc. Natl Acad. Sci. USA*, **85**, 1487.

SAKURA, A., KANEI-ISHII, C., NAGASE, T., NAKAGOSHI, H., GONDA, T.J. & ISHII, S. (1989). Delineation of three functional domains of the transcriptional activator encoded by the c-*myb* proto-oncogene. *Proc. Natl Acad. Sci. USA*, **86**, 5758.

SAP, J., MUNOZ, A., DAMM, K. & 5 others (1986). The c-*Erb*A protein is a high-affinity receptor for thyroid hormone. *Nature*, **324**, 635.

SASSONE-CORSI, P., LAMPH, W.W., KAMPS, M. & VERMA, I.M. (1988). fos- associated cellular P39 is related to nuclear transcription factor AP-1. *Cell*, **54**, 553.

SCHEIDEREIT, C., CROMLISH, J.A., GERSTER, T. & 4 others (1988). A human lyphoid-specific transcription factor that activates immunoglobulin genes is a homeobox protein. *Nature*, **336**, 551.

SCHUH, R., AICHER, W., GAUL, U. & 8 others (1986). A conserved family of nuclear proteins containing structural elements of the finger proteins encoded by Kruppel, a *Drosophila* segmentation gene. *Cell*, **47**, 1025.

SCHLIEF, R. (1988). DNA binding by proteins. *Science*, **241**, 1182.

SCHWAB, M., ALITALO, K., KLEMPNAUER, K.-H. & 6 others (1983). Amplified DNA with limited homology to myc cellular oncogene is shared by human neuroblastoma cell lines and a neuroblastoma tumour. *Nature*, **305**, 245.

SCOTT, M.P. & CARROLL, S.B. (1987). The segmentation and homeotic gene network in early *Drosophila* development. *Cell*, **51**, 689.

SHEER, D., LISTER, T.A., AMESS, J. & SOLOMON, E. (1985). Incidence of the 15q + ;17q − chromosome translocation in acute promyelocytic leukaemia (APL). *Br. J. Cancer*, **52**, 55.

SHEPARD, J.C., MCGINNIS, W., CARRASCO, A.E., DEROBERTIS, E.M. & GEHRING, W.J. (1984). Fly and frog homeodomains show homology with yeast mating type regulatory proteins. *Nature*, **310**, 70.

SHIMA, E.A., LEBEAU, M.M., MCKEITHAN, T.W. & 6 others (1986). Gene encoding the α chain of the T cell receptor is moved immediately downstream of c-*myc* in a chromosomal 8:14 translocation in a cell from a human T cell leukemia. *Proc. Natl Acad. Sci. USA*, **83**, 3439.

SMEAL, T., ANGEL, P., MEEK, J. & KARIN, M. (1989). Different requirements for formation of *jun:jun* and *jun:fos* complexes. *Genes Dev.*, **3**, 2091.

STAVNEZER, E., BRODEUR, D. & BRENNAN, L. (1989). The v-*ski* oncogene encodes a truncated set of c-*ski* coding exons with limited sequence and structural relatedness to v-*myc*. *Mol. Cell. Biol.*, **9**, 4038.

STEPHENS, R.M., RICE, N.R., HIEBSCH, R.R., BOSE, H.R. & GILDEN, R.V. (1983). Nucleotide sequence of v-*rel*: the oncogene of the reticuloendotheliosis virus. *Proc. Natl Acad. Sci. USA*, **80**, 6229.

STRUHL, K. (1988). The JUN oncoprotein, a vertebrate transcription factor, activates transcription in yeast. *Nature*, **334**, 649.

STURM, R.A. & HERR, W. (1988). The POU domain is a bipartite DNA-binding structure. *Nature*, **336**, 601.

TANAKA, M. & HERR, W. (1990). Differential transcriptional activation by *oct*-1 and *oct*-2: interdependent activation domains induce *oct*-2 phosphorylation. *Cell*, **60**, 375.

THOMPSON, C.C. & EVANS, R.M. (1989). *Trans*-activation by thyroid hormone receptors: functional parallels with steroid hormone receptors. *Proc. Natl Acad. Sci. USA*, **86**, 3494.

THOMPSON, E.A. (1989). Glucocorticoid inhibition of gene expression and proliferation of murine lymphoid cells *in vitro*. *Cancer Res.*, **49**, 2259s.

VAN BEVEREN, C., VAN STRAATEN, F., CURRAN, T., MULLER, R. & VERMA, I.M. (1983). Analysis of FBJ-MuSV provirus and c-*fos* (mouse) gene reveals that viral and cellular *fos* gene products have different carboxy termini. *Cell*, **32**, 1241.

VARMUS, H. & BISHOP, J.M. (1986). (eds). Biochemical mechanisms of oncogene activity: proteins encoded by oncogenes. *Cancer Surv.*, **5**, 153.

VILARES, R. & CABRERA, C.V. (1987). The achaete-scute gene complex of *D. melanogaster*: conserved domains in a subset of genes required for neurogenesis and their homology to *myc*. *Cell*, **50**, 415.

VINSON, C.R., SIGLER, P.B. & MCKNIGHT, S.L. (1989). Scissors grip model for DNA recognition by a family of leucine zipper proteins. *Science*, **246**, 911.

VOGT, P.K., BOS, T.J. & DOOLITTLE, R.F. (1987). Homology between the DNA binding domain of the GCN4 regulatory protein of yeast and the carboxy terminal region of a protein coded for by the oncogene *jun*. *Proc. Natl Acad. Sci. USA*, **84**, 3316.

VOGT, P.K. & TJIAN, R. (1988). *Jun*: a transcriptional regulator turned oncogenic. *Oncogene*, **3**, 3.

WASYLYK, B., WASYLYK, C., FLORES, P., BEGUE, A., LEPRINCE, D. & STEHELIN, D. (1990). The c-*ets* proto-oncogenes encode transcription factors that cooperate with c-*fos* and c-*jun* for transcriptional activation. *Nature*, **346**, 191.

WATSON, D.K., MCWILLIAMS, M.J., LAPIS, P., LAUTENBERGER, J.A., SCHWEINFEST, C.W. & PAPAS, T.S. (1988). Mammalian *ets*-1 and *ets*-2 genes encode highly conserved proteins. *Proc. Natl Acad. Sci. USA*, **85**, 7862.

WEINBERGER, C., THOMPSON, C.C., ONG, E.S., LEBO, R., GRUOL, D.I. & EVANS, R.M. (1986). The c-*Erb*A gene encodes a thyroid hormone receptor. *Nature*, **324**, 641.

WEISSMAN, B.E., SAXON, P.J., PASQUALE, S.R., JONES, G.R., GEISER, A.G. & STANBRIDGE, E.J. (1987). Introduction of a normal human chromosome 11 into a Wilms' tumour cell line controls its tumorigenic expression. *Science*, **236**, 175.

WESTON, K. & BISHOP, J.M. (1989). Transcriptional activation by the v-*myb* oncogene and its cellular progenitor c-*myb*. *Cell*, **58**, 85.

WILLIAMS, D.L., LOOK, A.T., MELVIN, S.L. & 4 others (1984). New chromosomal translocation correlate with specific immunophenotypes of childhood acute lymphoblastic leukaemia. *Cell*, **36**, 101.

WONG, A.J., BIGNER, S.H., BIGNER, D.D., KINZLER, K.W., HAMILTON, S.R. & VOGELSTEIN, B. (1987). Increased expression of the epidermal growth factor receptor gene in malignant gliomas is invariably associated with gene amplification. *Proc. Natl Acad. Sci. USA*, **84**, 6899.

WRIGHT, W.E., SASSON, D.A. & LIN, V.K. (1989). Myogenin, a factor regulating myogenesis, has a domain homologous to MyoD. *Cell*, **56**, 607.

ZERIAL, M., TOSCHI, L., RYSECK, R.-P., SCHUERMANN, M., MULLER, R. & BRAVO, R. (1989). The product of a novel growth factor activated gene, *fos*B, interacts with JUN proteins enhancing their DNA binding activity. *EMBO J.*, **8**, 805.